# Physician agreement on the diagnosis of sepsis in the intensive care unit: estimation of concordance and analysis of underlying factors in a multicenter cohort

**DOI:** 10.1186/s40560-019-0368-2

**Published:** 2019-02-21

**Authors:** Bert K. Lopansri, Russell R. Miller III, John P. Burke, Mitchell Levy, Steven Opal, Richard E. Rothman, Franco R. D’Alessio, Venkataramana K. Sidhaye, Robert Balk, Jared A. Greenberg, Mark Yoder, Gourang P. Patel, Emily Gilbert, Majid Afshar, Jorge P. Parada, Greg S. Martin, Annette M. Esper, Jordan A. Kempker, Mangala Narasimhan, Adey Tsegaye, Stella Hahn, Paul Mayo, Leo McHugh, Antony Rapisarda, Dayle Sampson, Roslyn A. Brandon, Therese A. Seldon, Thomas D. Yager, Richard B. Brandon

**Affiliations:** 1 Division of Infectious Diseases and Clinical Epidemiology, Intermountain Medical Center, Murray, UT 84107 USA; 2 Division of Infectious Diseases, University of Utah School of Medicine, Salt Lake City, UT 84132 USA; 3 Division of Pulmonary and Critical Care Medicine, Intermountain Medical Center, Murray, UT 84107 USA; 4 Division of Respiratory, Critical Care, and Occupational Pulmonary Medicine, University of Utah School of Medicine, Salt Lake City, UT 84132 USA; 5 Brown University, Providence, RI 02912 USA; 6 Johns Hopkins University School of Medicine, Baltimore, MD 21205 USA; 7 Rush Medical College and Rush University Medical Center, Chicago, IL 60612 USA; 8 Loyola University Medical Center, Maywood, IL 60153 USA; 9 Grady Memorial Hospital and Emory University School of Medicine, Atlanta, GA 30303 USA; 10 Northwell Healthcare, New Hyde Park, NY 11042 USA; 11 Immunexpress Inc, 425 Pontius Avenue North, Suite 430, Seattle, WA 98109 USA

**Keywords:** Sepsis, Diagnosis, Inter-observer agreement, Intensive care

## Abstract

**Background:**

Differentiating sepsis from the systemic inflammatory response syndrome (SIRS) in critical care patients is challenging, especially before serious organ damage is evident, and with variable clinical presentations of patients and variable training and experience of attending physicians. Our objective was to describe and quantify physician agreement in diagnosing SIRS or sepsis in critical care patients as a function of available clinical information, infection site, and hospital setting.

**Methods:**

We conducted a post hoc analysis of previously collected data from a prospective, observational trial (*N* = 249 subjects) in intensive care units at seven US hospitals, in which physicians at different stages of patient care were asked to make diagnostic calls of either SIRS, sepsis, or indeterminate, based on varying amounts of available clinical information (clinicaltrials.gov identifier: NCT02127502). The overall percent agreement and the free-marginal, inter-observer agreement statistic kappa (*κ*_free_) were used to quantify agreement between evaluators (attending physicians, site investigators, external expert panelists). Logistic regression and machine learning techniques were used to search for significant variables that could explain heterogeneity within the indeterminate and SIRS patient subgroups.

**Results:**

Free-marginal kappa decreased between the initial impression of the attending physician and (1) the initial impression of the site investigator (*κ*_free_ 0.68), (2) the consensus discharge diagnosis of the site investigators (*κ*_free_ 0.62), and (3) the consensus diagnosis of the external expert panel (*κ*_free_ 0.58). In contrast, agreement was greatest between the consensus discharge impression of site investigators and the consensus diagnosis of the external expert panel (*κ*_free_ 0.79). When stratified by infection site, *κ*_free_ for agreement between initial and later diagnoses had a mean value + 0.24 (range − 0.29 to + 0.39) for respiratory infections, compared to + 0.70 (range + 0.42 to + 0.88) for abdominal + urinary + other infections. Bioinformatics analysis failed to clearly resolve the indeterminate diagnoses and also failed to explain why 60% of SIRS patients were treated with antibiotics.

**Conclusions:**

Considerable uncertainty surrounds the differential clinical diagnosis of sepsis vs. SIRS, especially before organ damage has become highly evident, and for patients presenting with respiratory clinical signs. Our findings underscore the need to provide physicians with accurate, timely diagnostic information in evaluating possible sepsis.

**Electronic supplementary material:**

The online version of this article (10.1186/s40560-019-0368-2) contains supplementary material, which is available to authorized users.

## Impact statement/at a glance commentary

The differential diagnosis of sepsis vs. systemic inflammatory response syndrome (SIRS) in intensive care unit (ICU) patients remains challenging. We studied physician agreement in patients admitted to ICUs for the task of performing this differential diagnosis. Our findings suggest that uncertainty in this diagnosis has multifactorial causes: physician training and experience; availability of relevant clinical information (i.e., clinical microbiology tests, antigen- or molecular-based pathogen detection tests, and radiology); and identification of the source of infection (if known or present). We observed the least agreement between or among physicians for diagnosing sepsis in patients with respiratory clinical signs. Our findings underscore the need for objective diagnostics to be applied at the earliest possible time for critically ill patients suspected of infection.

## Background

Diagnosis of sepsis remains a challenge for myriad reasons [1]. A physician may first begin to suspect sepsis in the early stages of the disease, before organ damage is evident, when clinical signs can be either absent, varied, or clinically indistinguishable from systemic inflammation due to non-infectious causes. Further, for patients suspected of sepsis, clinical microbiology tests may be negative but when positive often require two or more days to produce actionable results. These microbiologic data suffer from significant numbers of false-positives and false-negatives when attempting to identify the actual microbial cause of sepsis [2, 3]. Early diagnosis of sepsis is important because intervention could potentially have the greatest patient benefit early in the disease course [4, 5]. However, inaccuracies in early sepsis diagnosis could have significant potential consequences for patients including excessive use of empiric, broad-spectrum antibiotics, inappropriate management, long-term morbidity, or death [6–8].

Sepsis definitions have evolved significantly over the last 30 years [9–12]. The early Sepsis-1 definition [9] described a septic syndrome which included clinical evidence of an infection along with fever or hypothermia, tachypnea, tachycardia, and evidence of impaired organ perfusion or function as manifested by either altered mentation, hypoxemia, elevated plasma lactate, or oliguria. Only 45% of patients with septic syndrome in this study were blood culture positive. Following from this, Bone et al. [10] introduced a definition for systemic inflammatory response syndrome (SIRS) and defined sepsis as an infection or suspected infection leading to SIRS. In 2001, a Sepsis-2 definition included at least two SIRS criteria and a suspected or confirmed infection [11]. In the Sepsis-3 definition, SIRS criteria are deemed to not be ideal clinical markers for sepsis, especially since it has been shown in a large study that 12% of patients with confirmed sepsis do not show clinical signs of SIRS [13] and since SIRS criteria are present in many hospitalized patients without infection [14]. Instead, the Sepsis-3 definition relies on an increase of the sequential organ failure assessment (SOFA) score of 2 points or more to determine organ dysfunction associated with a higher risk of in-hospital mortality. Thus, even after 30 years of effort, sepsis definitions continue to evolve. Singer et al. [12] state “there are, as yet, no simple and unambiguous clinical criteria, or biological, imaging, or laboratory features that uniquely identify a septic patient.” The Sepsis-3 definition, while seemingly an improvement in operational terms, nonetheless has been criticized because it shifts the emphasis to organ dysfunction, thus de-emphasizing detection and intervention at earlier stages when the disease is most easily treated [15–17].

In the absence of an unambiguous definition of sepsis and highly accurate diagnostic tools, physicians rely on their own clinical skill set and experience to diagnose sepsis. However, it has been shown that clinical diagnosis of sepsis upon admission to ICU corresponds poorly with post hoc presence of infection [18] and that agreement among physicians within specific sepsis diagnostic subgroups varies considerably [19].

Our study objective was to further identify and delineate factors contributing to the difficulty of the early diagnosis of sepsis, by quantifying the agreement between physicians for sepsis diagnosis in a cohort of adult patients prospectively enrolled in a multi-site clinical trial [20]. There are three ways in which our study extends previous work in this area: (1) it quantifies the physician agreement in sepsis diagnosis as a function of timing and availability of clinical information, physician training/experience level, hospital location, and infection site; (2) it offers a multivariate analysis of the observed heterogeneity within the patient group having indeterminate diagnoses; and (3) it employs machine learning methods to search for significant combinations of clinical variables that could explain a split of SIRS patients into those receiving vs. not receiving systemic antibiotics. An early version of this work has been presented in the form of an abstract [21].

## Methods

### Study definitions

Sepsis-3, the third international consensus definition of sepsis [12], represents a significant change from previous definitions of sepsis, giving emphasis to organ dysfunction and dysregulated immune response to infection. However, the present study employed the earlier Sepsis-2 definitions of systemic inflammatory response syndrome (SIRS) and sepsis [11] for several reasons: (1) the study was designed and initiated before the Sepsis-3 definition was published; (2) the Sepsis-3 definition is not particularly helpful to physicians for determining whether or not a patient suspected of sepsis has an infection, particularly in the early stages before organ dysfunction is evident [22]; and (3) in the USA, the Centers for Medicare and Medicaid Services (CMS) still use the Sepsis-2 definition for regulatory and reimbursement purposes; thus this definition is still used in practice [23].

In the present study, SIRS was defined as the presence of two or more clinical signs of systemic inflammation [10, 11] combined with (1) no apparent site of infection identified at admission or during hospitalization; (2) an alternative, non-infectious explanation for the signs of systemic inflammation; or (3) no microbial pathogen identified by culture, serologic, or antigen-based testing. Sepsis was defined by the presence of two or more signs of systemic inflammation, combined with a site of infection identified at admission or during early hospitalization, either with pathogen identification (“definite” infection) or without (“probable” infection). A diagnosis of “indeterminate” was defined as the combination of (1) two or more signs of systemic inflammation; (2) a possible non-infectious cause; and (3) a potential site of infection or an organism identified by culture, serologic, or antigen-based testing from a non-sterile site.

### Study cohort

We conducted a post hoc analysis of patient data from a prospective observational study [20], entitled *V*alidation of septic gene *E*xpressio*N U*sing *S*eptiCyte (VENUS) which was conducted at the Intermountain Medical Center (IMC), Murray, UT (*N* = 125) and LDS Hospital (LDSH), Salt Lake City, UT (*N* = 4) between April 2013 and April 2014. This study had a supplement, conducted between March 2016 and August 2016, that enrolled 120 additional patients from five academic institutions in major US metropolitan areas: Johns Hopkins Hospital (JHH), Baltimore, MD (*N* = 39); Rush University Medical Center (RUMC), Chicago, IL (*N* = 37); Loyola University Medical Center (LUMC), Maywood, IL (*N* = 11); Northwell Healthcare (NH), Long Island, NY (*N* = 26); and Grady Memorial Hospital (GMH), Atlanta, GA (*N* = 7). The VENUS and VENUS supplement patients (*N* = 249) are herein together referred to as the “USA Cohort.” The VENUS and VENUS supplement cohorts were recruited under the same study protocol (clinicaltrials.gov identifier: NCT02127502) and employed the same inclusion and exclusion criteria. The VENUS and VENUS supplement cohorts were recruited as the US component of a parent study, involving a total of 447 patients that was powered to evaluate the diagnostic performance of a new molecular test, SeptiCyte LAB, for distinguishing sepsis from SIRS in adult critical care patients [20].

### Patient inclusion and exclusion criteria

#### Inclusion criteria

Adults (18–89 years) were considered for enrollment in the study if they displayed an accumulation (usually in the ED) of two or more clinical signs of systemic inflammation (temperature > 38 °C or < 36 °C, heart rate > 90 beat/min, tachypnea > 20/min or PaCO_2_ < 32 mm Hg, white blood cell count > 12,000/mm^3^ or < 4000/mm^3^, or > 10% immature neutrophils) in the 24-hour period prior to being considered for enrollment. Enrollment occurred within 24 h of admission to ICU. Informed consent was obtained for each subject, either directly or through a legally authorized representative.

#### Exclusion criteria

Subjects were excluded if consent was not obtained, if bacterial infection was suspected but no microbiology cultures were collected, if admitted to ICU for ≥ 24 h before consent or study enrollment, or if undergoing elective cardiac surgery with an expected ICU stay of < 24 h.

### Clinical diagnostic methods

Clinical diagnoses at each study site were performed using four methods that differed with respect to the extent of training and experience of the evaluators and also to the timing and the amount of clinical information available (Table 1).

**Table 1:** Clinical diagnostic methods

	Classification method			
	1. Initial assessment: attending physician	2. Initial assessment: site investigator	3. Discharge assessment: site investigators	4. External RPD
By:	Attending physician (*N* = 1)	Site investigator (*N* = 1)	Site investigators (*N* = 2)	External expert panel (*N* = 3)
When:	Within 24 h of admission to ICU	Within 48 h of admission to ICU (nearly always within 24 h)	At discharge from ICU	Following discharge from ICU
Using:	Clinical signs at admission and basic laboratory and radiology results	Clinical signs at admission and basic laboratory and radiology results	• Retrospective data (first 24 h in ICU) • Microbiology • Non-culture based pathogen detection results • Radiology	• Retrospective data (first 24 h in ICU) • Microbiology • Non-culture-based pathogen detection results • Radiology • Retrospective discharge assessment
Output	SIRS/indeterminate/sepsis	SIRS/indeterminate/sepsis	SIRS/indeterminate/sepsis	SIRS/indeterminate/sepsis
Adjudication	None	None	If site investigators do not agree, then 3rd independent physician’s vote taken	Full agreement = SIRS or sepsis 2/3 agreement = SIRS or sepsis No agreement = Indeterminate

#### Initial assessment (attending physician)

An initial clinical assessment was performed by the attending physician using clinical data available within 24 h of ICU admission as part of routine care, independent of the present study. The attending physician was required to make a diagnostic call of infection status (none, possible, probable, or definite) [18, 19, 24]. When one of the site investigators was the admitting physician, this investigator’s initial clinical impression served as the attending physician impression. This assessment was made before culture results became available and represents routine clinical practice.

Note: in the subsequent analysis of data from the attending physicians, an infection status of “none” was assigned to the SIRS category, an infection status of “possible” was assigned to the indeterminate category, and an infection status of “probable” or “definite” was assigned to the sepsis category. This converted the attending physician assessments into a format consistent with the assessments performed by the site investigators and external panelists (below).

#### Initial assessment (site investigator)

An initial assessment was also performed by one of the two site investigators who was not the treating physician. A site investigator never provided both attending and investigator initial clinical impressions. Site investigators included pulmonary intensivists and infectious diseases specialists. The site investigator’s assessment was made using clinical data available within 24 h of admission and did not include an independent physical examination or culture results. Site investigators were required to make one of the following diagnostic calls: no infection (SIRS), possible infection (indeterminate), probable infection (sepsis), or definite infection (sepsis). This method aimed to most closely resemble a case referred to an experienced infectious disease or critical care specialist, who was then required to make an initial clinical impression based on currently available clinical information.

#### Discharge assessment (site investigators’ consensus)

A discharge evaluation was performed independently by the two site investigators who examined each subject’s medical record from admission to hospital discharge. This assessment used only the portion of the complete medical record that was relevant to the systemic inflammation observed during the initial 24–48-h period in ICU. Clinical evaluation and workup was determined by the treating physician. Tests that the site investigators reviewed to establish a discharge diagnosis included clinical notes, radiographic data, operative notes, pathology reports, culture results, and/or results of antigen- or molecular-based pathogen detection tests. Cultures were collected based on the suspected site of infection (e.g., blood, urine, wound, respiratory tract, etc.). Positive test results were interpreted in conjunction with the clinical scenario to establish a diagnosis. Site investigators were required to make one of the following diagnostic calls: no infection (SIRS), possible infection (indeterminate), probable infection (sepsis), or definite infection (sepsis). Disagreement in diagnostic calls between the two site investigators triggered an independent review by an equally qualified adjudicator. Using this method, a disagreement between all three evaluators, or a diagnostic call of “possible” by all three evaluators, was classified as indeterminate. This method aimed to establish a reference call made by on-site investigators (“local reference”).

#### Retrospective physician diagnosis (RPD)

An independent panel of three external expert physicians performed a final discharge assessment for each enrolled patient, which served as the gold standard (“external reference”) for the diagnosis of sepsis or SIRS. Each panel member was a senior physician with expertise across intensive care medicine, emergency medicine, and/or infectious diseases but was not involved in the patient’s care and did not have access to the full patient medical records. All patient case review forms (CRFs) containing the study subjects’ collection site and clinical information in a standardized format were forwarded to each panel member. The panel members were also given access to the consensus discharge evaluations made by the site investigators. The diagnostic call options were no infection (SIRS), possible infection (indeterminate), probable infection (sepsis), and definite infection (sepsis). This method aimed to match previously published studies [18, 19, 24] and was viewed as the gold standard for diagnosing sepsis because it incorporated all the earlier clinical data and judgments, drew upon the RPD panelists’ broad expertise, and helped to ensure a consistent interpretation of clinical data across study sites.

### Diagnostic comparisons

To quantify the agreement between pairs of clinical evaluations, we computed the overall percent agreement and also two versions (fixed-marginal and free-marginal) of the inter-observer agreement statistic kappa (denoted by the symbols *κ*_fixed_ and *κ*_free_, respectively). Details are provided in Additional file 1. The following letters (A–K) are used throughout the manuscript and its supplements, to denote the following comparisons:

A. Initial assessment (attending physician) versus initial assessment (site investigator), in an attempt to delineate the influence of different levels of physician training and experience

B. Initial assessment (attending physician) versus consensus discharge assessment (site investigators), in an attempt to compare the accuracy of an attending physician’s initial impression with the locally determined reference diagnosis (local reference)

C. Initial assessment (attending physician) versus consensus RPD (external panelists) to compare accuracy of an attending physician’s initial impression with the expert reference diagnosis (external reference)

D. Initial assessment (site investigator) versus consensus discharge assessment (site investigators), in an attempt to delineate the influence of diagnostic test results on clinical impression

E. Initial assessment (site investigator) versus consensus RPD (external panelists), in an attempt to delineate the influence of physician training and experience level, and of diagnostic test results

F. Consensus discharge assessment by site investigators versus consensus RPD (external panelists), in an attempt to understand the variability that may occur in a panel of experienced physicians

G, H, I. Comparisons between individual RPD evaluations in an attempt to delineate or quantify the baseline level of disagreement (“diagnostic noise”) inherent in the de facto gold standard

J. Comparisons between the discharge assessments of site investigators

K. Unanimous Agreement, representing the highest degree of diagnostic certainty. We defined “unanimous” to mean that the site investigators’ consensus discharge assessment and the individual evaluations by the three external RPD panelists were in complete agreement regarding the diagnosis of SIRS or sepsis. If the agreement was less than unanimous, then an indeterminate call was made under this evaluation method.

Note that comparison (F) was expected to display a relatively high level of agreement because the external panel was given access to the site investigators’ consensus discharge evaluations; thus comparison (F) provides a realistic upper bound on expected agreement values.

Agreement between various clinical evaluations was quantified by the overall percent agreement and also by two variants of the inter-observer kappa statistic. The commonly used Cohen’s kappa or fixed-marginal variant *κ*_fixed_ [25, 26] was unsuitable for those comparisons involving small numbers of samples; instead, we used Randolph’s free-marginal multirater kappa *κ*_free_ [27], which is well-defined and robust in this context. Further details on these statistics are provided in Additional file 1. A line data file is provided in Additional file 2.

### Statistical and bioinformatics analyses

Differences between proportions were evaluated for significance using a two-proportion Z-test (http://www.socscistatistics.com/tests/ztest/Default2.aspx) when sample sizes were large (n*p > 5). For small sample sizes (n*p < 5), an N-1 chi-square test was used instead (https://measuringu.com/ab-cal/). Two-tailed tests were employed. The Kolmogorov-Smirnov test was used to check for significance of differences between cumulative distributions, using an applet available at www.physics.csbsju.edu/stats/KS-test.html. The significance (*p* value) of the D statistic was checked with the online calculator at http://home.ubalt.edu/ntsbarsh/Business-stat/otherapplets/pvalues.htm#rkstwo.

We performed logistic regression followed by receiver operating characteristic (ROC) curve analysis, in an attempt to identify factors that could stratify the indeterminate subjects or identify underlying similarities to SIRS or sepsis archetypes. To perform these calculations, an online logistic regression calculator (http://statpages.info/logistic.html) and an online ROC curve calculator (http://www.rad.jhmi.edu/jeng/javarad/roc/JROCFITi.html) were used.

We also analyzed the SIRS patient subgroup (106/249; 42.6% of total, as defined by unanimous agreement) by machine learning methods, in an attempt to identify factors that could explain the treat/no treat decision. We tried several approaches. (1) From the line data file, we selected a pool of twelve multi-valued parameters (N.SIRS, ICU.LoS, Hospital.LOS, Age, HeartRate.Max, HeartRate.Min, APACHE.Score, Mean.Art.Pressure.Min, WBC.Max, WBC.Min, Glucose.Max, Glucose.Min) and eight binary-valued (+/−) parameters (culture.blood, culture.drain, culture.pus, culture.respiratory, culture.skin, culture.sputum, culture.urine, culture.other.contaminants). Then, using either logistic regression or Random Forests [28, 29] and a recursive feature elimination process [30, 31], we searched for classifiers (combinations of the above parameters) that could discriminate between treated and untreated SIRS patients. (2) We performed a linguistic analysis of single words and word pairs in the “physician comment” field of the case report, using a Random Forests approach, in a further attempt to discriminate between treated and untreated SIRS patients. (3) Finally, we pooled the most informative clinical and demographic parameters, words, and word-pairs from above, and repeated the recursive feature elimination using either logistic regression or Random Forests.

## Results

### Description of study cohorts

Our study enrolled 249 patients with demographic and clinical characteristics presented in Table 2. With the unanimous diagnostic method, which is arguably the most accurate because it requires perfect agreement between the consensus discharge diagnosis and all three RPD panelists, 106/249 (42.6%) of subjects were diagnosed as SIRS, 69/249 (27.8%) were diagnosed as sepsis, and 74/249 (29.7%) of subjects were assigned an indeterminate status. Pneumonia was the most commonly identified infection site (42/249; 16.9%). Patients with an indeterminate diagnosis, or who were unanimously diagnosed with sepsis, were older and had longer stays in ICU and hospital and also had a higher median Acute Physiology and Chronic Health Evaluation (APACHE) score compared to patients diagnosed as SIRS.

**Table 2:** Demographic and clinical characteristics of the study cohort (*N*=249). Comparator = unanimous method, meaning that the site investigators’ consensus discharge assessment and the individual evaluations by the three external RPD panelists were in complete agreement, regarding the diagnosis of SIRS or sepsis. If the agreement was less than unanimous, then an indeterminate call was made

Parameter category	Parameter	SIRS (*n* = 106)	Sepsis (*n* = 69)	Indeterminate (*n* = 74)	*p* value^1^
Demographics	Age: median (IQR)	54 (40–65)	60 (47–67)	64 (53–75)	0.002
	Sex: female	51 (48%)	30 (44%)	36 (49%)	0.79
	White	68 (64%)	41 (59%)	53 (72%)	0.30
	Black	30 (28%)	16 (23%)	16 (22%)	0.55
	Asian/East Indian	2 (2%)	6 (9%)	2 (3%)	0.06
	Hispanic	4 (4%)	5 (7%)	3 (4%)	0.54
	Other or unrecorded	2 (2%)	1 (1%)	0	0.51
Blood culture result	1. No blood culture done or blood culture negative	100 (94%)	11 (16%)	66 (89%)	< 0.001
	2. Blood culture positive	3 (3%)	29 (42%)	4 (5%)	< 0.001
	3. Gram positive	3 (3%)	13 (19%)	3 (4%)	< 0.001
	4. Gram negative	0	11 (16%)	1 (1%)	< 0.001
	5. Mixed Gram pos/neg	0	5 (7%)	0	0.001
	6. Fungus	0	0	0	NA
Infection site	Respiratory tract (non-lung)	0	3 (4%)	4 (5%)	0.06
	Lung (pneumonia)	3 (3%)	15 (22%)	24 (32%)	< 0.001
	Abdominal	0	10 (14%)	2 (3%)	< 0.001
	Urinary tract	0	8 (12%)	3 (4%)	0.001
	Other site	0	21 (30%)	4 (5%)	< 0.001
	Not identified	103 (97%)	12 (17%)	37 (50%)	< 0.001
Clinical parameters, outcome	Days in hospital: median (IQR)	3 (2–5)	8 (5–14)^3^	6 (4–9)	< 0.001
	Days in ICU: median (IQR)	2 (1–2)	3 (2–5)	2 (1–4)	0.002
	Antibiotics given in ICU	60 (57%)	68 (99%)	68 (92%)	< 0.001
	APACHE score: median (IQR)^2^	54 (29–84)	76 (46–95)^3^	82 (48–103)^4^	< 0.001
	SOFA score: median (IQR)	4 (2–7)^6^	5 (4–10)^5^	6 (4–8)^7^	0.02
	Mortality	6 (6%)	9 (13%)^3^	9 (12%)	0.18

*Abbreviations*: *ANOVA* analysis of variance, *APACHE* Acute Physiology and Chronic Health Evaluation, *ICU* intensive care unit, *IQR* inter-quartile range, *NC* not calculated, *neg* negative, *pos* positive, *RPD* retrospective physician diagnosis, *SOFA* sequential organ failure assessment

^1^For distributions (like age), the *p* value is derived from ANOVA. For categorical variables such as sex, the *p* value is derived from a three-sample test for equality of proportions without continuity correction. *p* values derived from small samples should not be considered definitive.

^2^APACHE score, as calculated at different clinical sites (site, version, available to RPD panelists): IMH III yes; LDSH III yes; JHH III no; NH IV no; RUMC II yes; LUMC III no; GMH III no.

^3^68/69 sepsis patients with data recorded

^4^73/74 indeterminate patients with data recorded

^5^55/69 sepsis patients with data recorded

^6^70/106 SIRS patients with data recorded

^7^60/74 indeterminate patients with data recorded

### Comparison of initial assessment to later assessments

**Fig. 1:**
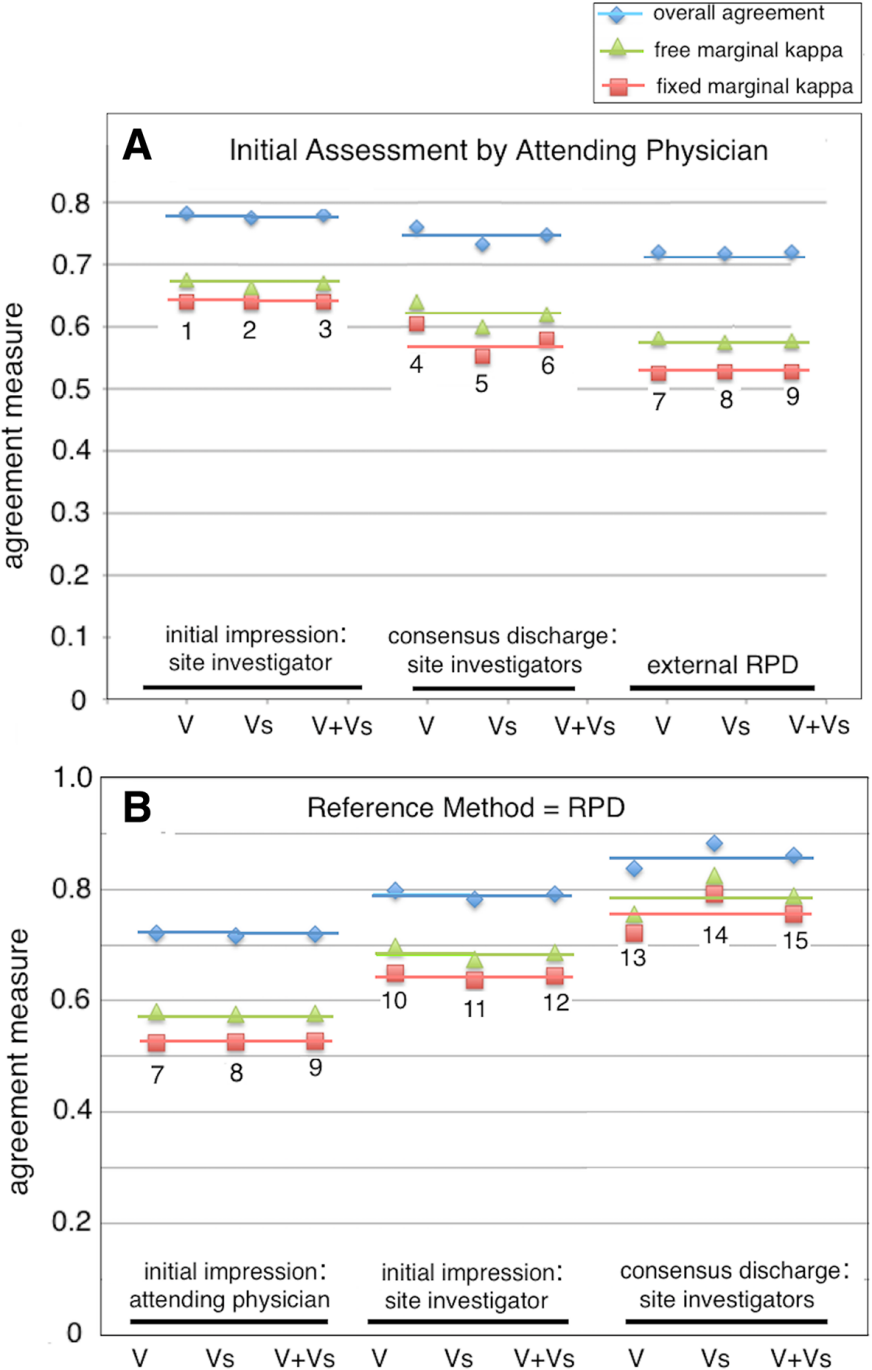
Agreement between diagnostic methods. **a** Comparisons 1, 2, and 3: initial assessment by attending physician vs. initial assessment by site investigator. Comparisons 4, 5, and 6: initial assessment by attending physician vs. discharge assessment by site investigators. Comparisons 7, 8, and 9: initial assessment by attending physician vs. external RPD. Agreement with the initial assessment by attending physician decreases (fixed-marginal kappa *κ*_fixed_ 0.64 ➔ 0.58 ➔ 0.53) as more diagnostic information becomes available, as physician training and experience increases, and as time pressure to make a diagnostic call decreases. **b** Comparisons 7, 8, and 9: initial assessment by attending physician vs. external RPD. Comparisons 10, 11, and 12: initial assessment by site investigator vs. external RPD. Comparisons 13, 14, and 15: consensus discharge assessment by site investigators vs. external RPD. The numerals and symbols in this figure have the following meanings: 1, 4, 7, 10, and 13: VENUS cohort (V; 129 subjects); 2, 5, 8, 11, and 14: VENUS supplemental cohort (Vs; 120 subjects); 3, 6, 9, 12, 15: VENUS + VENUS supplemental cohorts (V + Vs; 249 subjects); blue diamonds = overall agreement; green triangles = free-marginal kappa *κ*_free_; red squares = fixed-marginal kappa *κ*_fixed_

We compared the initial assessment by the attending physician to the initial and discharge assessments by the site investigator(s) and also to the external discharge RPD. Figure 1a summarizes the percent overall agreement and inter-observer kappa values for these comparisons. The fixed-marginal kappa (*κ*_fixed_) between initial clinical impressions by the attending physician and site investigator was moderate at 0.64 (95% CI 0.56–0.72). The value of *κ*_fixed_ between the initial assessment by the attending physician and the retrospective discharge assessment by the site investigators was marginally lower at 0.58 (95% CI 0.49–0.67). The value of *κ*_fixed_ between initial assessment by the attending physician and the external RPD was lower still at 0.53 (95% CI 0.44–0.62). Thus, we found an apparent trend of decreasing fixed-marginal kappa (*κ*_fixed_ 0.64 ➔ 0.58 ➔ 0.53) between the initial impression made by treating physicians and final diagnoses as determined by the local and expert panels. The free-marginal kappa values (*κ*_free_) followed the same trend but were slightly higher than their fixed-marginal counterparts.

We also compared the initial assessment by the attending physician and the initial and discharge assessments by the site investigator(s) to the external RPD gold standard (Fig. 1b). We found that the reference diagnosis as determined by the RPD panel agreed better with initial impressions made by the site investigators (*κ*_fixed_ 0.64) than with the initial impressions of the attending physician (*κ*_fixed_ 0.53). We also observed that the consensus discharge impression of site investigators had a moderate and greatest agreement with the consensus of the external RPD panelists (*κ*_fixed_ 0.76). The free-marginal kappa values (*κ*_free_) followed the same trend but were slightly higher than their fixed-marginal counterparts (Fig. 1b).

A difference in agreement between the initial and later assessments is reflective of the influences that cumulative test results and response to empiric therapy may have on clinical impressions. We also categorized the degree of risk and theoretical impact that initial misdiagnosis would have on treatment decisions and patient outcome (Table 3 and Additional file 3). An increased apparent risk for poor outcome was considered to occur if a patient was initially assessed as SIRS or indeterminate but was then reclassified later as indeterminate or sepsis. By this measure, 21 patients (8.4%) had an increase in apparent risk with four (1.6%) initially thought to have SIRS but ultimately determined to be septic by the local panel diagnosis. This difference was greater when compared to the external RPD (Table 3 and Additional file 3**)**.

**Table 3:** Analysis of Reclassification Events

Reclassification	Number (%) reclassified: attending physician ➔ discharge evaluation by site investigators (Additional file 3: Figure S3–1)	Number (%) reclassified: attending physician ➔ RPD (Additional file 3: Figure S3–2)	Change in apparent in risk profile	Potential consequence of erroneous initial classification
SIRS to sepsis	4 (1.6%)	6 (2.4%)	Low to high	Delayed antibiotic treatment, prolonged hospital stay, and increased morbidity and mortality
Indeterminate to sepsis	12 (4.8%)	22 (8.8%)	Medium to high	Possible delayed antibiotic treatment
SIRS to indeterminate	5 (2.0%)	5 (2.0%)	Low to medium	Possible delayed antibiotic treatment
Sepsis to SIRS	9 (3.6%)	9 (3.6%)	High to low	Excess antibiotic use
Indeterminate to SIRS	18 (7.2%)	16 (6.4%)	Medium to low	Possible excess antibiotic use
Sepsis to indeterminate	15 (6.0%)	12 (4.8%)	High to medium	Possible excess antibiotic use
Total	63 (25.3%)	70 (28.1%)		

### Stratification by infection site

**Fig. 2:**
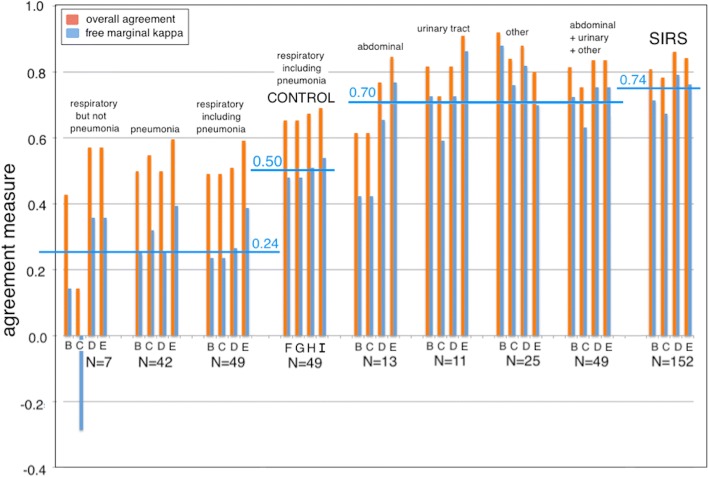
Plot of percent overall agreement and free-marginal kappa (*κ*_free_) for diagnostic method comparisons stratified by site of infection. For each infection site, the following comparisons were performed: (B) initial assessment by attending physician vs. consensus discharge assessment by site investigators; (C) initial assessment by attending physician vs. external RPD; (D) initial assessment by site investigator vs. consensus discharge assessment; and (E) initial assessment by site investigator vs. external RPD. As a control, the following comparisons were performed for respiratory infection samples including pneumonia (*N* = 49): (F) consensus discharge assessment vs. external RPD; (G) RPD panelists 1 vs. 2; (H) RPD panelists 1 vs. 3; (I) RPD panelists 2 vs. 3. Note: the number of subjects in the various categories add up to 250 (not 249) because the infection site for one sepsis case was diagnosed as both abdominal and pneumonia. “SIRS” indicates that no site of infection was identified. Horizontal blue bars indicate average values for the free-marginal kappa statistic, over the indicated comparisons

We examined the agreement between initial assessment and later diagnosis based on infection site. We stratified the cohort into the following categories of infection site: non-pneumonia respiratory, pneumonia, abdominal, urinary tract, other site, or not identified (Fig. 2). The category “not identified” was considered to be equivalent to a diagnosis of SIRS. This analysis employed the overall percent agreement and the free-marginal kappa (*κ*_free_) as appropriate for small sample sizes.

We observed that the lowest levels of diagnostic agreement between initial and later diagnoses were found for both categories of respiratory infection (pneumonia and non-pneumonia), with the free-marginal kappa (κ_free_) having a mean value of + 0.24 (range − 0.29 to + 0.39) for respiratory infections, compared to + 0.70 (range 0.42 to 0.88) for all other infections. For cases where no infection site could be identified (i.e., presumptive SIRS), the free-marginal kappa (*κ*_free_) had a mean value of 0.74 (range 0.67 to 0.79).

The low agreement between physicians for patients with respiratory infections could not be attributed solely to differences between initial and later diagnoses. This was shown by the comparisons labeled “control” in Fig. 2, in which agreement for respiratory infections was measured for the site investigators’ consensus discharge assessment vs. RPD (F), and between pairs of RPD panelists (G, H, I). These control comparisons for respiratory infection sources show a much lower degree of agreement (*κ*_free_ = 0.50) relative to comparisons involving other infection sources (*κ*_free_ = 0.70) or SIRS (*κ*_free_ = 0.74). Thus, sepsis cases of respiratory origin appear inherently difficult to diagnose, regardless of clinical data gathered during a patient’s ICU stay and regardless of the point at which the diagnosis is being attempted (ICU admission, ICU discharge, or external RPD). Additional details of the analysis of the patients with pneumonia or other respiratory infections are presented in Additional file 4.

We attempted to identify clinical variables that could help to explain the differences in inter-observer agreement between patients with respiratory infections versus other types of infection. We stratified patients into the following two subgroups with respect to infection site: respiratory (pneumonia + non-pneumonia) versus non-respiratory (abdominal + urinary + other). We then used statistical tests (*t* test or 2-proportions *z* test) to ascertain if clinical variables could be identified that displayed significantly different values between these two subgroups (Table 4**)**. The respiratory infection subgroup had a significantly higher percentage of patients diagnosed as indeterminate, with lower procalcitonin (PCT) values, lower maximum body temperature (T Max), and higher mean arterial pressure (MAP) compared to patients in the non-respiratory infection subgroup.

**Table 4:** Parameters that vary significantly between different infection site subgroups

Parameter	Mean ± SD (non-respiratory)	Mean ± SD (respiratory)	*p* value^1^
Number in group	48	49	
Overall agreement	0.85 ± 0.05	0.62 ± 0.08	6.0E-09
Free-marginal *κ*	0.77 ± 0.07	0.43 ± 0.12	6.8E-09
No. of indeterminates (identified by discharge consensus assessment)	6/48 = 12.5%	15/49 = 30.6%	0.030
Lowest MAP	53.9 ± 16.4	64.4 ± 17.1	0.003
Max temperature	38.3 ± 1.0	37.7 ± 0.8	0.002
Min temperature	36.0 ± 0.70	36.4 ± 0.7	0.03
Log_2_ PCT	2.58 ± 3.10	− 0.46 ± 3.82	0.001

Patients with identified sites of infection (*N* = 97) were stratified into the following subgroups: non-respiratory (abdominal + urinary + other; *N* = 48) and respiratory (pneumonia + non-pneumonia; *N* = 49). Parameters that varied significantly (*p* < 0.05) between these two groups were identified by statistical testing (*t* test for continuous parameters; two-proportions *z*-test for categorical parameters)

^1^Two-tailed *t* test for all parameters except for overall agreement and number of indeterminates, for which a two-proportion *z*-test was run instead (www.vassarstats.net)

### Stratification by hospital

We stratified our cohort with respect to different hospitals and then evaluated the agreement among different diagnostic methods. This analysis employed the free-marginal kappa (*κ*_free_) as appropriate for small sample sizes. We observed significant differences among hospitals with respect to the level of agreement among methods (Additional file 5). The cause(s) of the differences among hospitals were not obvious. Patients from some hospitals could have been more difficult to diagnose, due to differences in presentation or severity of clinical signs. Alternatively, the explanation might reside in differences in training or institutional practices.

### Analysis of indeterminate diagnoses

**Fig. 3:**
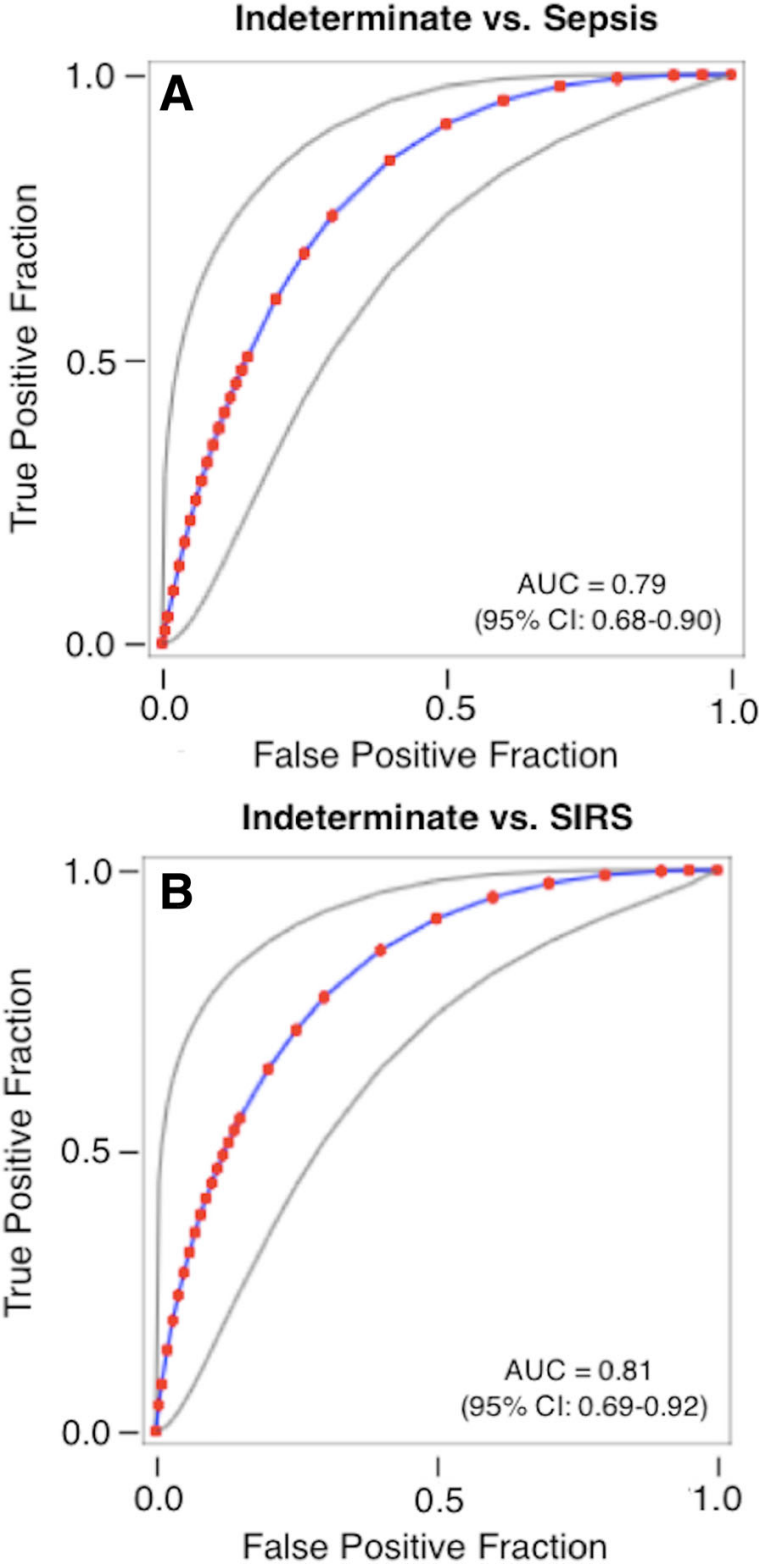
Logistic regression analysis to distinguish indeterminates from patients with either sepsis or SIRS. **a** Logistic regression model for sepsis vs. indeterminates. The model used consensus discharge diagnosis by the site investigators as the comparator and analyzed 64 septic patients and 23 indeterminates. The predictor variable is given by the following equation: *y* = 0.4249 + 0.3672 * SeptiScore + 0.1232 * WBC.Max − 0.0245 * WBC.Min − 0.0269 * MAP.Max. This equation gives AUC = 0.79 (95% CI 0.68–0.90) in ROC curve analysis. **b** Logistic regression model for SIRS vs. indeterminates. The model used consensus discharge diagnosis by the site investigators as the comparator and analyzed 73 SIRS patients and 15 indeterminates. The predictor variable is given by the following equation: *y* = 3.1742–0.2548 * log2 PCT − 0.3913 * SeptiScore. This equation gives AUC = 0.81 (95% CI 0.69–0.92) in ROC curve analysis. Additional file 6 provides further details of the analysis. Abbreviations: AUC, area under curve; MAP.Max, maximum mean arterial blood pressure; PCT, procalcitonin; ROC, receiver operating characteristic curve; WBC.Max, maximum white blood cell count; WBC.Min, minimum white blood cell count

The classification methods of Table 1 allowed for patients to have a final designation of “indeterminate” when physicians were unable to make a clear classification of either sepsis or SIRS, or when retrospective panel designations were contradictory. For patients classified as indeterminate, we found that certain clinical variables were able to discriminate indeterminates from either sepsis or SIRS but not simultaneously from both groups. However, there were no composite clinical variables that clearly achieved a three-way discrimination between SIRS, sepsis, and indeterminate groups. The two ROC curves of Fig. 3 illustrate the results of a logistic regression analysis. In panel a, a logistic combination of the variables SeptiScore, WBC.Max, WBC.Min, and MAP.Max differentiated 64 septic patients from 23 indeterminates with an AUC of 0.79 (95% CI 0.68–0.90). Similarly, a logistic combination of log_2_ PCT and SeptiScore differentiated 73 SIRS patients from 15 indeterminates with an AUC of 0.81 (95% CI 0.69–0.92). Thus, it appears that a major difficulty in resolving indeterminates into either the SIRS or sepsis category reflects an inherent clinical uncertainty which is not resolved by patient signs and symptoms and diagnostic tests. Further details on this analysis are provided in Additional file 6**.**

### Antibiotic use

**Fig. 4:**
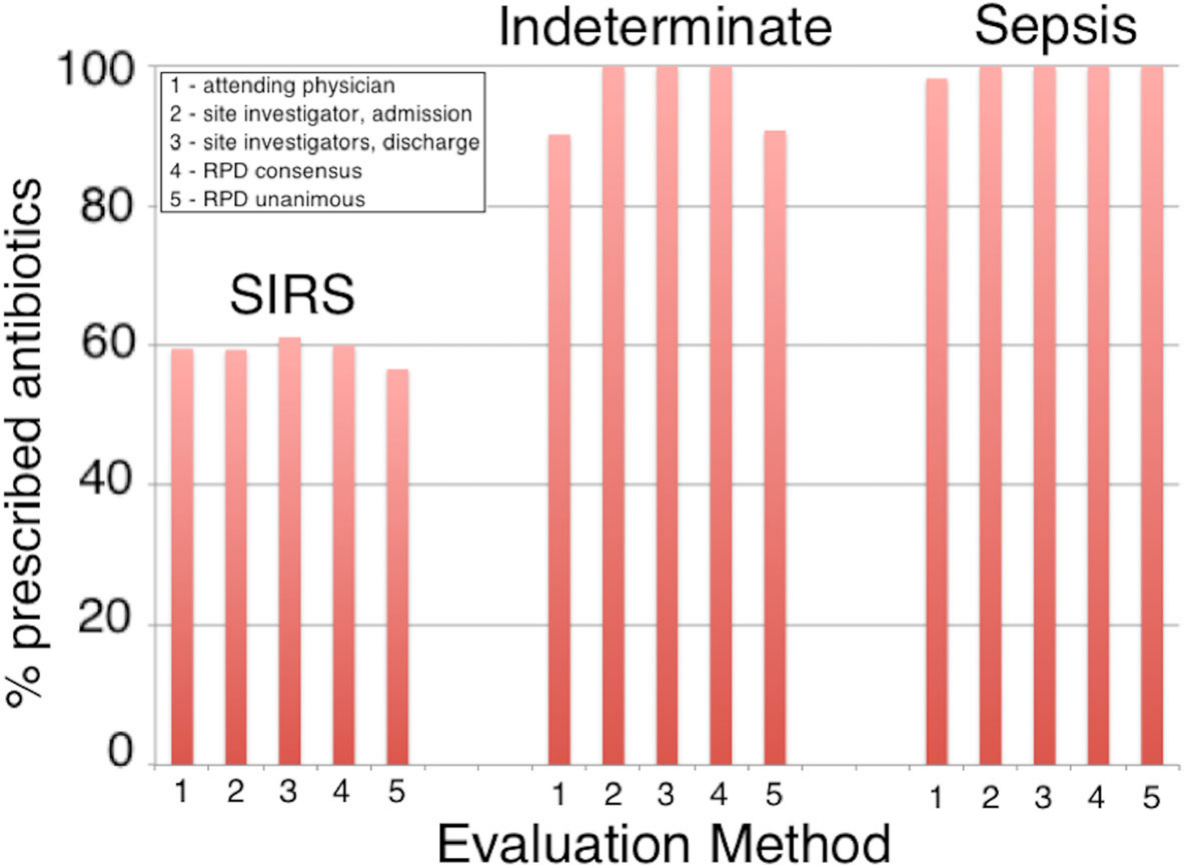
Analysis of subjects treated with therapeutic antibiotics as a function of diagnosis, evaluation method, and cohort: fraction of subjects treated. The case report forms indicated whether or not particular patients were treated with therapeutic antibiotics. A diagnosis of SIRS, indeterminate, or sepsis was made by (1) attending physician at admission, (2) site investigator at admission, (3) site investigators’ consensus at discharge, (4) consensus RPD, or (5) unanimous RPD

**Fig. 5:**
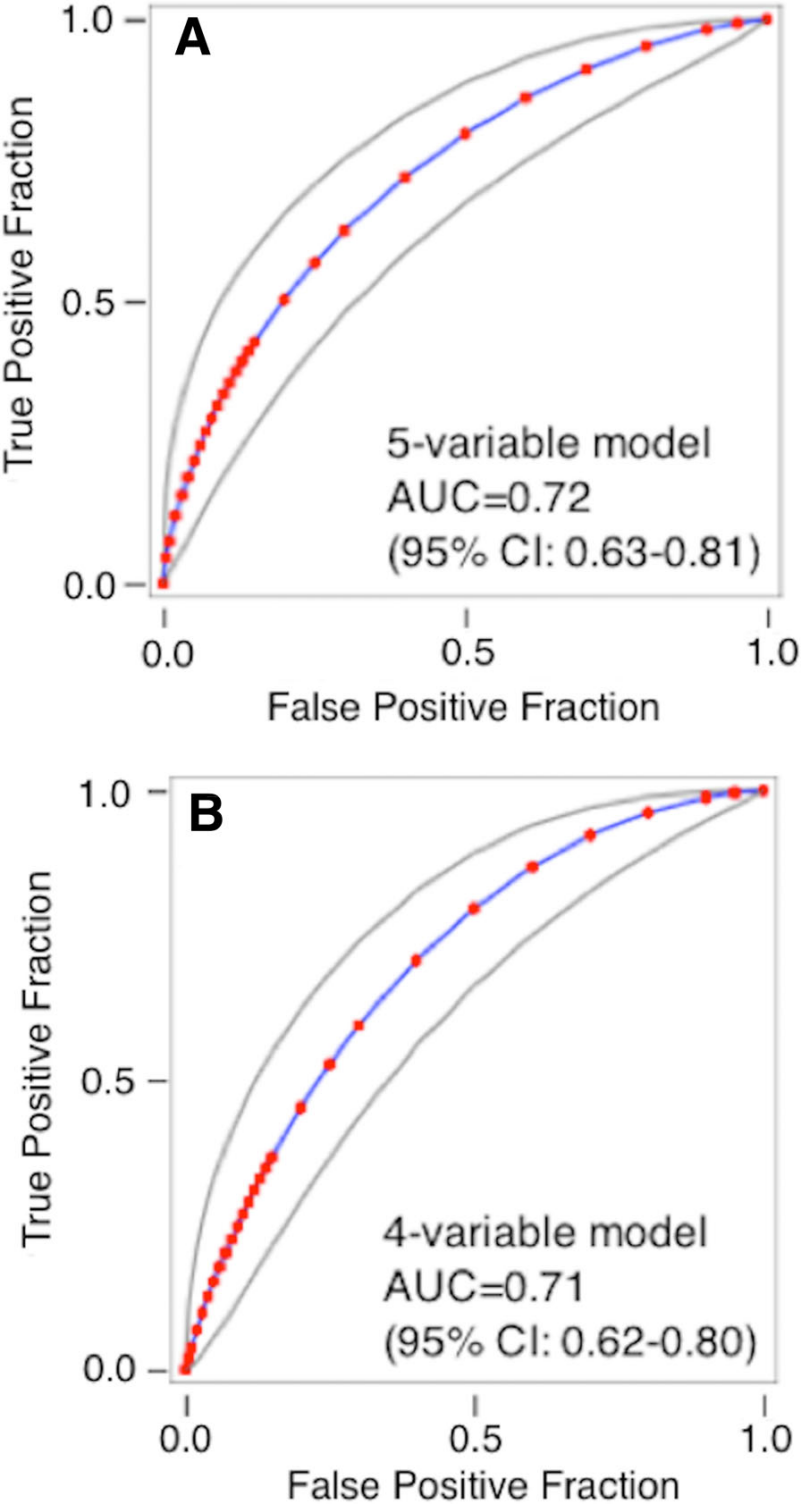
Analysis of subjects treated with therapeutic antibiotics as a function of diagnosis, evaluation method, and cohort**:** logistic regression models. **a** Discrimination of SIRS patients who were treated vs. not treated with therapeutic antibiotics, using a five-variable logistic model (*y* = − 17.8210 − 0.0200 * MAP.Min + 0.0128 * HR.Max + 0.4540 * Temp.Max + 0.0906 * Hospital.LoS + 0.2472 * N.SIRS). The model gave AUC 0.72 (95% CI 0.63–0.81) in ROC curve analysis. **b** Discrimination of SIRS patients who were treated vs. not treated with therapeutic antibiotics using a four-variable logistic model (y = − 16.5106 - 0.0239 * MAP.Min + 0.0125 * HR.Max + 0.4372 * Temp.Max + 0.2386 * N.SIRS). The model gave AUC 0.71 (95% CI 0.62–0.80) in ROC curve analysis. Additional file 7 provides further details. Abbreviations: AUC, area under curve; Hospital.LoS, length of stay in hospital; HR.Max, maximum heart rate; MAP.Min, minimum mean arterial blood pressure; N.SIRS, number of SIRS criteria met; ROC, receiver operating characteristic curve; Temp.Max, maximum core temperature; WBC.Max, maximum white blood cell count; WBC.Min, minimum white blood cell count

Antibiotics were administered to 60/106 (56.6%) of patients unanimously diagnosed as SIRS, 78/78 (100.0%) patients unanimously diagnosed as sepsis, and 59/65 (90.8%) of indeterminate patients (Fig. 4). We searched for individual factors underlying the physicians’ decisions to give antibiotics to only a subset of SIRS patients. This analysis is described in Table 5 and Additional file 7. At least five clinical and demographic parameters (low MAP, tachycardia, fever, number of SIRS criteria (N.SIRS), and increased hospital length of stay (H.LoS)) showed some ability individually (*p* < 0.05 in *t* test) to distinguish between SIRS patients who were either treated or not treated with antibiotics. The four variables MAP, tachycardia, fever, and N.SIRS, either with or without the increased H.LoS, were combined in logistic regression, and the resultant predictor was able to achieve a partial discrimination (AUC 0.71–0.72), as shown in Fig. 5a, b.

**Table 5:** Test for the ability of clinical parameters to distinguish between SIRS patients who did or did not receive therapeutic antibiotics

Parameter	Number of datapoints available^1^		*p* value
	Patients receiving antibiotics	Patients not receiving antibiotics	
MAP.Min	75	52	0.003
HR.Max	78	52	0.007
Temp.Max	78	52	0.011
Hospital LoS	78	52	0.014
N.SIRS	78	52	0.022
SOFA	54	36	0.043
Log_2_ PCT	51	34	0.054
APACHE	78	51	0.080
WBC.Min	78	51	0.153
pH	34	17	0.183
Age	78	52	0.272
Race: non-white	23/78 (29.5%)	20/52 (38.5%)	0.286
Race: white	55/78 (70.5%)	32/52 (61.5%)	0.286
WBC.Max	78	51	0.352
Glucose.Max	68	46	0.406
SeptiScore	78	52	0.413
Sex: female	35/78 (44.9%)	27/52 (51.9%)	0.430
Sex: male	43/78 (55.1%)	25/52 (48.1%)	0.430
Lactate	39	17	0.473
MAP.Max	58	42	0.694
ICU LoS	78	52	0.749
Temp.Min	74	51	0.760
HR.Min	78	52	0.960

Diagnosis of SIRS (*N* = 130) was by consensus RPD. Parameters are listed on the basis of decreasing significance (two-tailed *p* value) as evaluated either by *t* test (for continuous variables) or by a two-proportions *z*-test (www.vassarstats.net) for categorical variables

*Abbreviations*: *Glucose.Max* maximum blood glucose concentration, *HR.Max* maximum heart rate, *HR.Min* minimum heart rate, *ICU LoS* length of stay in ICU (days), *MAP.Max* maximum mean arterial blood pressure, *MAP.Min* minimum mean arterial blood pressure, *N.SIRS* number of SIRS criteria met, *Temp.Max* maximum core temperature, *Temp.Min* minimum core temperature, *WBC.Max* maximum white blood cell count, *WBC.Min* minimum white blood cell count

^1^No imputation of missing values was performed

These results appear to suggest that physicians may rely on clinical and demographic parameters, perhaps embodied in intuitive judgments, as the basis of antibiotic treatment decisions in these SIRS patients. To further explore this possibility, we performed a more sophisticated machine-learning analysis, in which we used recursive feature elimination, logistic regression, Random Forests, and textual analysis of the “physicians comments” field in the case reports, to identify combinations of parameters that could discriminate between treated and untreated patients in the SIRS group. Results are described in Additional file 7. The conclusion we reached is that no combination of clinical, demographic, or textual variables was able to distinguish between treated vs. untreated patients in the SIRS group with an AUC greater than approximately 0.7.

## Discussion

Despite the evolution and improvement of sepsis definitions over the past three decades [9–12], clinicians still have difficulty identifying patients who are becoming septic, especially early in the process before organ dysfunction has become severe. Previous attempts have been made to describe the difficulty in diagnosing sepsis in quantitative terms, through measuring agreement between physician diagnoses and sepsis definitions [32–34], between physicians and nurses [35], between ED physicians and hospitalists [36], or between physicians on a post hoc basis [19]. In the last-named comparison, using all available clinical information, inter-observer agreement varied considerably, with lowest levels of agreement for ventilator-associated pneumonia. The reported variation in agreement on a post hoc basis demonstrates the challenges faced by physicians in accurate sepsis diagnosis at the time of patient presentation, when clinical information is limited.

Following from this earlier work, we conducted a post hoc analysis of previously collected data from a prospective, observational trial of 249 adult subjects who fulfilled ≥ 2 SIRS criteria. The subjects were recruited from the ICUs of seven hospitals in the USA. We sought to determine the degree to which existing clinical approaches were able to distinguish sepsis from non-infectious causes of SIRS. Physicians with different levels of training and experience (attending physicians, site investigators, and external expert panelists) were asked to make diagnostic calls of either SIRS, sepsis, or indeterminate, based on varying amounts of clinical information available. Agreement between evaluators was quantified using overall percent agreement and the free-marginal and fixed-marginal variants of the inter-observer agreement kappa statistic (*κ*_free_ and *κ*_fixed_, respectively).

Our study extends the previous work on this topic because it quantifies differences in sepsis diagnosis based on timing and availability of clinical information, physician training, hospital location, and/or infection site. It also attempts to identify significant clinical variables underlying the observed heterogeneity within the group of patients with indeterminate diagnoses. Finally, it employs machine learning methods to attempt to identify clinical variables that could explain why, of the patients diagnosed as SIRS, some were prescribed antibiotics and some were not.

We identified the following key factors affecting the inter-observer agreement values: (1) the type and amount of clinical information available (initial impression upon ICU admission vs. discharge impression or RPD) and (2) whether the infection was respiratory in origin or arose in some other body site. In general, physician agreement was moderate (*κ*_free_ ~ 0.7) for diagnosing either SIRS or sepsis due to non-respiratory infections. The agreement for diagnosing sepsis due to respiratory infections was significantly lower (*κ*_free_ ~ 0.3). This last finding appears to be consistent with other reports from the literature [19, 37–39].

An important aspect of our study is the capture of the initial admitting impression and its comparison to later diagnosis at ICU discharge or by expert panel. We believe the admission classification of the attending physician best represents real world decision-making process, and realistically embodies the expected clinical variability in clinical judgement which is often subjective in nature early in a disease course. We found that a physician’s initial clinical impression often disagreed with the final diagnosis, with a tendency to over diagnose sepsis early, as 30% of those reclassified were initially determined to be septic by the treating physicians.

We found low inter-observer agreement for differentiating SIRS vs. sepsis in cases of respiratory infections (Fig. 3 and Additional file 4), consistent with other reports from the literature [19, 37–39]. Earlier studies have shown a poor predictive value of the use of clinical signs and symptoms in detecting radiographic pneumonia [40]. Similarly, chest x-ray has also been shown to be inaccurate with poor inter-observer agreement in diagnosing pneumonia when compared to quantitative respiratory cultures collected by protected brush specimens [41]. Clearly, accurate diagnostic tests that improve a physician’s ability to distinguish between pneumonia, viral causes of respiratory tract infection, and non-infectious inflammatory processes are needed.

A noteworthy observation from our study is that ~ 60% of patients ultimately classified as having SIRS nonetheless were given systemic antibiotics. An extensive bioinformatics analysis failed to identify variables, or combinations of variables, that could definitively discriminate between SIRS patients that did or did not receive systemic antibiotics: the best combination of variables produced a ROC AUC of only ~ 0.7 between these groups. The early use of antibiotics in patients with SIRS suggests that physicians, when confronted with critically ill patients displaying systemic signs and symptoms that could indicate the presence of sepsis—and prior to establishing a definitive diagnosis—will take vigorous measures to initiate antibiotics early, which is a practice supported by the scientific literature, national guidelines, and the Surviving Sepsis Campaign [42–44]. The fact that antibiotic use was so frequent in patients who did not have infection highlights the need for better strategies to reduce the burden and duration of unnecessary antibiotic use.

Our study has several limitations. First, due to practical constraints, it was not possible for the attending physician and the site investigator to provide the admission evaluation concurrently. Some component of the disagreement between the initial assessments of the attending physician and the site investigator may therefore have been due to differences in the (time-dependent) availability of information used to make this early assessment, rather than to differences in data interpretation used to assign infection likelihoods. However, generally the initial assessments of the attending physician and site investigator were performed within hours of each other, and therefore drew from very similar available data in the electronic medical record. In all cases, both opinions were rendered before microbiological results became available. Thus there was little opportunity for differences in assessment by attending physician and site investigator to derive from the arrival of definitive (i.e., microbiological culture) evidence in the intervening time period. Second, the external RPD panelists had access to the site investigators’ retrospective discharge assessment for each patient, which means there was no complete independence between these two assessments. Third, by design, we did not power the underlying study for stratification with the exception of the different cohorts (VENUS, VENUS supplemental) and pneumonia as an infection source. There could be other strata-specific diagnostic discordances that were not readily observable because of the limited size of our cohorts. In the case of pneumonia, which was targeted a priori, we observed a marked and significant decrease in diagnostic agreement in this stratum compared to other infection sources. Lung conditions are diverse in etiology, and an additional study powered to provide insight into this important patient group could be valuable.

We believe the true level of diagnostic uncertainty encountered in clinical practice may be underestimated by the percent overall agreement and inter-observer agreement statistics used in this study. Factors not addressed in our analysis that could contribute to additional diagnostic uncertainty include: (1) inherent subjectivity in result interpretation by physicians, (2) unknown institutional (site) differences including the use of decision support tools (see Additional file 5), (3) demographic or clinical heterogeneity, and (4) uncertainty in SIRS and sepsis definitions. It is interesting that there were 8/249 (3.2%) truly ambiguous cases, for which the discharge evaluation by the adjudicator matched neither evaluation by the site investigators. We believe that these eight truly ambiguous cases most likely represent a background level of “inherent diagnostic noise” that could not be eliminated from the study. Removal of these patients did not significantly change the study’s conclusions (not shown).

## Conclusions

In a post hoc analysis of data from a prospectively enrolled, multicenter cohort, we found that the initial clinical impressions made on admission to ICUs moderately agreed with the final clinical diagnosis as determined by experienced site investigators and by an external expert panel of specialists. The highest level of disagreement was observed in patients with respiratory tract symptoms. Antibiotic use was widespread and not always indicated, with over 50% of patients initially thought to have SIRS receiving empiric treatment on admission. Our findings further underscore the need for improved diagnostic tests that can be applied early in a patient’s hospitalization to better guide therapeutic decisions that include the withholding of antibiotics.

## Additional files

Additional file 1: Comparison of Different Kappa Statistics. **Figure S1–1.** Sample size dependence of the *κ*_free_/*κ*_fixed_ ratio. Data were taken from the USA cohort, stratified by hospital collection site (Additional file 5). **Figure S1–2.** Different kappa statistics, plotted as a function of overall percent agreement. Data were generated from the stratification analysis with respect to hospital collection site (Additional file 5). (PDF 215 kb)

Additional file 2: Line Data File. (XLS 425 kb)

Additional file 3: Analysis of Reclassification Events. **Figure S3–1.** Reclassification events between the initial impression by the attending physician and the consensus discharge evaluation by site investigators. **Figure S3–2.** Reclassification events between the initial impression by the attending physician and the RPD. (PDF 333 kb)

Additional file 4: Patients with Respiratory Infections. **Figure S4–1.** Cumulative distributions of the overall percent agreement statistic for respiratory infections vs. non-respiratory infections + SIRS, calculated from Tables S4–1 and S4–2. The Kolmogorov-Smirnov test indicated a highly significant difference  (*p* < 0.0001). **Figure S4–2.** Cumulative distributions of the free marginal kappa statistic (*κ*_free_) for respiratory infections vs. non-respiratory infections + SIRS, calculated from Tables S4–1 and S4–2. The Kolmogorov-Smirnov test indicated a highly significant difference (*p* < 0.0001). **Figure S4–3.** Measured classification discordance in the VENUS + VENUS supplement cohorts (*N* = 49). (A) Comparison of initial evaluations by attending physician and site investigator (67.3% overall agreement; *κ*_free_ = 0.51). (B) Comparison of the attending physician’s initial evaluation and site investigators’ discharge evaluation (49.0% overall agreement; *κ*_free_ = 0.24). (C) Comparison of discharge assessments between site investigators (67.3% overall agreement; *κ*_free_ = 0.51). (D) Comparison of site investigators’ consensus discharge assessment and external RPD (65.3% overall agreement; *κ*_free_ = 0.48). **Figure S4–4.** Measured classification discordance in the VENUS + VENUS supplement cohorts without respiratory infections (*N* = 207). (A) Comparison of the initial evaluations of the attending physician and the site investigator (80.5% overall agreement; *κ*_free_ = 0.71). (B) Comparison of attending physician’s initial evaluation and site investigators’ consensus discharge evaluation (81.0% overall agreement; *κ*_free_ = 0.72). (C) Comparison of discharge assessments between site investigators (93.0% overall agreement; *κ*_free_ = 0.90). (D) Comparison of site investigators’ consensus discharge assessment and external RPD (91.0% overall agreement; *κ*_free_ = 0.86). **Figure S4–5.** Cumulative Distributions of the Indeterminate Vote Fraction, for patients suspected of pneumonia or non-pneumonia respiratory infections (*N* = 49) versus patients not suspected of these conditions (*N* = 200). **Table S4–1.** Pairwise comparisons: respiratory infections. **Table S4–2.** Pairwise comparisons: non-respiratory infections + SIRS (PDF 640 kb)

Additional file 5: Stratification by Hospital. **Figure S5–1.** Plot of overall agreement and *κ*_free_ for different diagnostic methods at different US hospitals. Values are plotted for (U) the entire USA Cohort (*N* = 249) and individually for different sub-cohorts: hospital #1 (*N* = 129), hospital #2 (*N* = 11), hospital #3 (*N* = 39), hospital #4 (*N* = 26), hospital #5 (*N* = 37), and (6) hospital #6 (*N* = 7). Note that (U) = hospitals #1 + 2 + 3 + 4 + 5 + 6. Orange bars: overall percent agreement. Blue bars: free-marginal kappa. The comparisons were as follows: (A) initial assessment by attending physician vs. initial assessment by site investigator; (B) initial assessment by attending physician vs. consensus discharge assessment by site investigators; (C) initial assessment by attending physician vs. RPD consensus; (D) initial assessment by site investigator vs. consensus discharge assessment by site investigators; (E) initial assessment by site investigator vs. RPD consensus; (F) consensus discharge assessment by site investigators vs. RPD consensus. **Table S5–1.** Parameters with significant differences (*p* < 0.05) between hospital subgroups (hospitals #2, 3) versus (hospitals #5, 6). These two US hospital subgroups displayed the least vs. greatest agreement between the initial diagnosis at admission and later discharge or retrospective diagnoses. (PDF 479 kb)

Additional file 6: Analysis of Indeterminates. **Figure S6–1.** ROC curve analysis. Panel A: discrimination of sepsis vs. indeterminate, using the variable MAP.Max. Panel B: discrimination of SIRS vs. indeterminate, using the variable Temp.Max. **Figure S6–2.** Logistic regression model for sepsis vs. indeterminates. The predictor variable is given by the following equation: *y* = 0.4249 + 0.3672 * SeptiScore + 0.1232 * WBC.Max − 0.0245 * WBC.Min − 0.0269 * MAP.Max. This equation gives AUC = 0.79 (95% CI 0.68–0.90) in ROC curve analysis. **Figure S6–3.** Logistic regression model for SIRS vs. indeterminates. The predictor variable is given by the following equation: *y* = 3.1742–0.2548 * log2 PCT − 0.3913 * SeptiScore. This equation gives AUC = 0.81 (95% CI 0.69–0.92) in ROC curve analysis. **Table S6–1.** Comparison of clinical parameters for patients classified as sepsis, SIRS, or indeterminate, when consensus discharge by site investigators is the comparator. Dataset = Venus + Venus supplement (*N* = 249). Mean ± SD values are indicated. Significance testing: 2-tailed *t* test for continuous variables with equal variances assumed (Excel); two-proportion *Z*-test for categorical variables (http://www.socscistatistics.com/tests/ztest/Default2.aspx). Variables that show significant (*p* < 0.05) differences between sepsis/indeterminate groups, or between SIRS/indeterminate groups, are highlighted in pink. **Table S6–2.** Logistic regression to discriminate indeterminates from sepsis. **Table S6–3.** Logistic regression to discriminate indeterminates from SIRS. **Table S6–4.** Summary of logistic regression analysis (PDF 558 kb)

Additional file 7: Analysis of Treated vs. Untreated SIRS Patients. **Figure S7–1.** Behavior of logistic regression models in ROC curve analysis. (A) Five variable model from Table S7–2, giving AUC = 0.72 (95% CI 0.63–0.81). (B) Four variable model from Table S7–3, giving AUC = 0.71 (95% CI 0.62–0.80). **Figure S7–2.** Machine learning attempts to identify combinations of clinical variables and demographic variables that discriminate between antibiotic treatment and no treatment, within the SIRS group. Recursive feature elimination was used, within a logistic regression (LR) or Random Forests (RF) model. **Figure S7–3.** Gini ranking of individual words in the “physician comments” field of the case report form, for SIRS patients. The ranking is based on contribution toward discriminating antibiotic-treated vs. untreated SIRS patients. Abbreviations: cxr, chest x-ray; dka, diabetic ketoacidosis; mri, magnetic resonance imaging. **Figure S7–4.** Gini ranking of word-pairs in the “physician comments” field of the case report form, for SIRS patients. The ranking is based on contribution toward discriminating antibiotic-treated vs. untreated SIRS patients. Abbreviation: chf, congestive heart failure. **Figure S7–5.** Machine learning attempt to identify combinations of clinical variables, demographic variables, words, and word-pairs that discriminate between antibiotic treatment and no treatment, within the SIRS group. Recursive feature elimination was used, within a logistic regression (LR) or Random Forests (RF) model. **Table S7–1.** Test for ability of clinical and demographic parameters to distinguish between SIRS patients who received (AB+) or did not receive (AB−) therapeutic antibiotics. Diagnosis was by consensus RPD. Parameters are listed in order of decreasing significance (2-tailed p-value) as evaluated either by Student’s *t* test, assuming equal variances in the two groups (for continuous variables), or by a 2-proportions *z*-test (www.vassarstats.net) for categorical variables. **Table S7–2.** Use of logistic regression, to discriminate between SIRS patients who were treated vs. not treated with antibiotics. Five variable model. **Table S7–3.** Use of logistic regression, to discriminate between SIRS patients who were treated vs. not treated with antibiotics. Four variable model. (PDF 550 kb)

Additional file 8: STROBE Checklist. (XLSX 14 kb)

## Abbreviations

ANOVA: Analysis of variance
APACHE: Acute Physiology and Chronic Health Evaluation
AUC: Area under curve
CI: Confidence interval
CRF: Case report form
ICU: Intensive care unit
IQR: Inter-quartile range
IRB: Institutional review board
LB: Lower bound
NC: Not calculated
ROC: Receiver operating characteristic (curve)
SD: Standard deviation
SI: Site investigator
SOFA: Sequential organ failure assessment
UB: Upper bound
*κ*_fixed_: Fixed-marginal inter-observer agreement statistic
*κ*_free_: Free-marginal inter-observer agreement statistic
