# Supplementary material for: Physician agreement on the diagnosis of sepsis in the intensive care unit: estimation of concordance and analysis of underlying factors in a multicenter cohort
Source: J Intensive Care. 2019 Feb 21;7:13. doi: 10.1186/s40560-019-0368-2 (PMC6383290; doi:10.1186/s40560-019-0368-2)
Supplement: Supplementary file 1 — Comparison of Different Kappa Statistics. Figure S1–1. Sample size dependence of the κfree/κfixed ratio. Data were taken from the USA cohort, stratified by hospital collection site (Additional file 5). Figure S1–2. Different kappa statistics, plotted as a function of overall percent agreement. Data were generated from the stratification analysis with respect to hospital collection site (Additional file 5). (PDF 215 kb) [file 40560_2019_368_MOESM1_ESM.pdf]

Physician Agreement on the Diagnosis of Sepsis in the Intensive Care Unit: Estimation of Concordance and Analysis of Underlying Factors in a Multicenter Cohort

Bert K. Lopansri, Russell R. Miller III, John P. Burke, Mitchell Levy, Steven Opal, Richard E. Rothman, Franco R. D'Alessio, Venkataramana K. Sidhaye, Robert Balk, Jared A. Greenberg, Mark Yoder, Gourang Patel, Emily Gilbert, Majid Afshar, Jorge P. Parada, Greg S. Martin, Annette M. Esper, Jordan A. Kempker, Mangala Narasimhan, Adey Tsegaye, Stella Hahn, Paul Mayo, Leo McHugh, Antony Rapisarda, Dayle Sampson, Roslyn A. Brandon, Therese A. Seldon, Thomas D. Yager, Richard B. Brandon

Supplement S1: Comparison of Different Kappa Statistics

Our study employed several different measures of agreement between evaluators: the percent overall agreement, Cohen's kappa, fixed marginal kappa (κ_{fixed}), and free marginal kappa (κ_{free}). It is necessary to understand the strengths and weaknesses of these various measures.

An important point is that kappa statistics of the "fixed marginal" type (including Cohen's kappa) assume that the evaluators are constrained with respect to the *proportions* of cases they are allowed to distribute across categories. For large datasets it can reasonably be assumed that evaluators of equivalent skill and experience level will tend to agree pretty well in the proportions of cases they assign to different categories. However, for small datasets, these proportions may be subject to large fluctuations that could lead to the paradoxical behavior of high agreement but low κ_{fixed}.

In contrast, kappa statistics of the "free marginal" type remove the constraint on the proportions of cases distributed across categories, and therefore are more suitable for the analysis of small datasets (Randolph, 2005; Gwet, 2014).

Methods

The % overall agreement, free-marginal kappa (κ_{free}), and fixed-marginal kappa (κ_{fixed}) were calculated using the the web applet described by Randolph (2005) and accessed at the following website: http://justusrandolph.net/kappa/

Cohen's kappa was calculated using the web applet provided by Professor Richard Lowry at the following website: http://vassarstats.net/kappa.html

Results

In the present paper, comparisons involving large datasets - e.g. the entire USA cohort (VENUS + VENUS Supplement) - may be analyzable with fixed-marginal kappa statistics. However, when stratification is performed and smaller subsets of data are produced, it will be more appropriate to analyze these with free-marginal kappa statistics.

The above points are clearly illustrated by **Figure S1-1**, in which the K_{free} / K_{fixed} ratio is calculated as a function of sample size. Data are taken from the stratification of the USA cohort with respect to hospital collection site (Supplement S5).

Figure S1-1: *Sample size dependence of the* κ_{free} / κ_{fixed} ratio*.* Data were taken from the USA cohort, stratified by hospital collection site (Supplement S5).

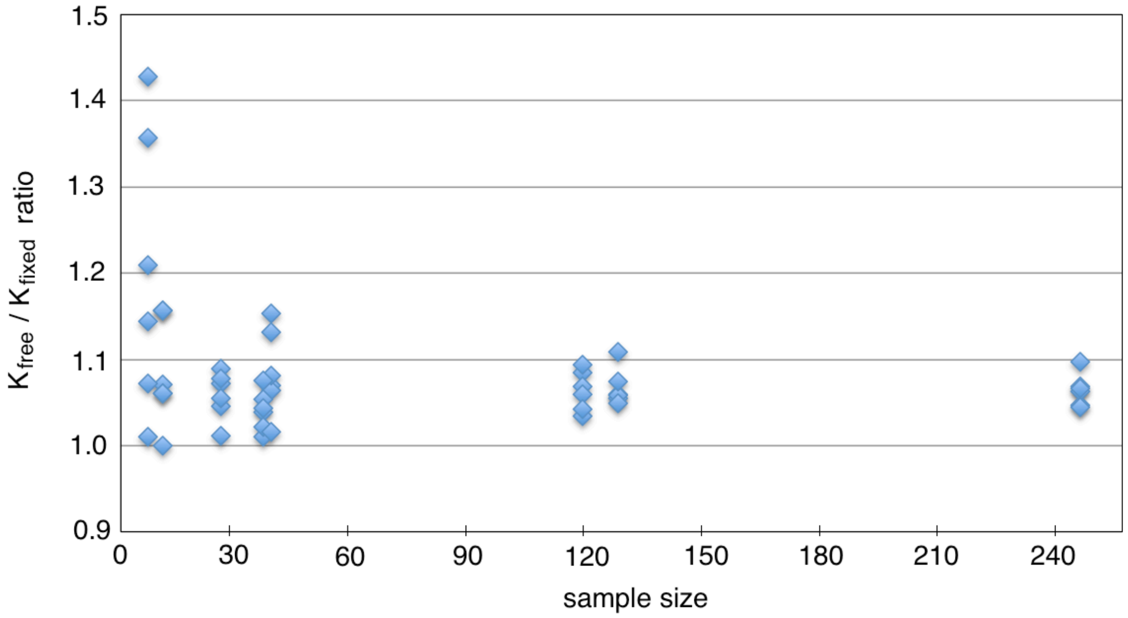

The above figure shows clearly that there are significant fluctuations in the κ_{free} / κ_{fixed} ratio for very small sample sizes. However, one should recognize that these occur as relatively minor perturbations on top of a larger pattern of regularity. In **Figure S1-2** we plot several different kappa statistics as a function of the overall % agreement. Again, data are taken from the stratification of the USA cohort with respect to hospital collection site (Supplement S5). The figure displays a fairly tight linear relationship.

Inspection of Figure S1-2 reveals the source of variability in the κ_{free} / κ_{fixed} ratio. This variability evidently is due to underlying fluctuations in the fixed-marginal / Cohen's components (black, orange points in Figure S1-2).

Figure S1-2: *Different kappa statistics, plotted as a function of overall % agreement.* Data were generated from the stratification analysis with respect to hospital collection site (Supplement S5).

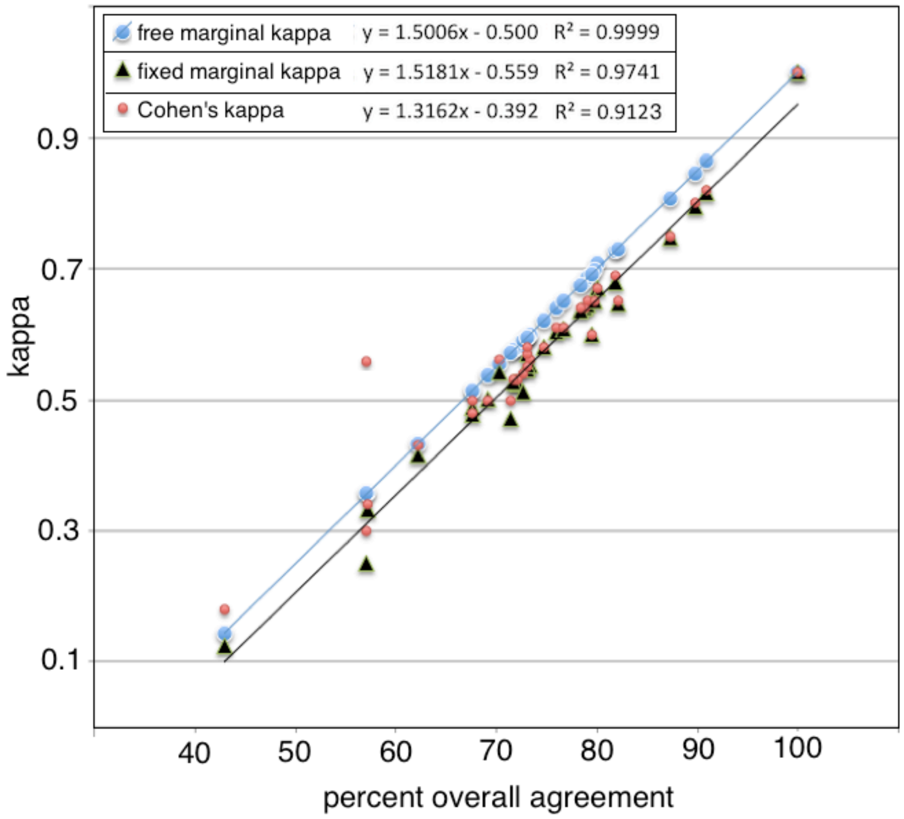

References

Gwet, K. (2014). Handbook of inter-rater reliability, 4th Edition. Advanced Analytics LLC, Gaithersburg MD. ISBN 978-0-9708062-8-4

Lowry, R. (2019) VassarStats website: Cohen's kappa calculator. Accessed on 10-jan-2018 at the following address: http://vassarstats.net/kappa.html

Randolph, J. J. (2005). Free-marginal multirater kappa: An alternative to Fleiss´ fixed-marginal multirater kappa. Paper presented at the Joensuu University Learning and Instruction Symposium 2005, Joensuu, Finland, October 14-15th, 2005. (ERIC Document Reproduction Service No. ED490661)
