# Supplementary material for: Physician agreement on the diagnosis of sepsis in the intensive care unit: estimation of concordance and analysis of underlying factors in a multicenter cohort
Source: J Intensive Care. 2019 Feb 21;7:13. doi: 10.1186/s40560-019-0368-2 (PMC6383290; doi:10.1186/s40560-019-0368-2)
Supplement: Supplementary file 3 — Analysis of Reclassification Events. Figure S3–1. Reclassification events between the initial impression by the attending physician and the consensus discharge evaluation by site investigators. Figure S3–2. Reclassification events between the initial impression by the attending physician and the RPD. (PDF 333 kb) [file 40560_2019_368_MOESM3_ESM.pdf]

Physician Agreement on the Diagnosis of Sepsis in the Intensive Care Unit: Estimation of Concordance and Analysis of Underlying Factors in a Multicenter Cohort

Bert K. Lopansri, Russell R. Miller III, John P. Burke, Mitchell Levy, Steven Opal, Richard E. Rothman, Franco R. D'Alessio, Venkataramana K. Sidhaye, Robert Balk, Jared A. Greenberg, Mark Yoder, Gourang Patel, Emily Gilbert, Majid Afshar, Jorge P. Parada, Greg S. Martin, Annette M. Esper, Jordan A. Kempker, Mangala Narasimhan, Adey Tsegaye, Stella Hahn, Paul Mayo, Leo McHugh, Antony Rapisarda, Dayle Sampson, Roslyn A. Brandon, Therese A. Seldon, Thomas D. Yager, Richard B. Brandon

Supplement S3: Analysis of Reclassification Events

This supplement expands upon the information contained in Table 3 of the text. We considered the reassignment of subjects between the initial evaluation by the attending physician, and the patient reevaluation at ICU discharge or RPD.

Results

Reclassification rates were analyzed for the USA cohort (VENUS + VENUS Supplement), between the initial evaluation by the attending physician, and the patient reevaluation at either ICU discharge or Retrospective Physician Diagnosis (RPD). We observed a reclassification rate of 25-28% as shown in **Figure S3-1** and **Figure S3-2**.

A patient's initial classification by the attending physician served as the basis for initial treatment and patient management decisions. If the initial classification was wrong, it follows that inappropriate or delayed treatments and patient management decisions may have been made. Thus, when patients are reclassified, their apparent risk profiles are altered. **Table S3-1** delineates some of the potential consequences of assumed incorrect initial classifications.

Figure S3-1: Reclassification events between the initial impression by the attending physician, and the consensus discharge evaluation by site investigators.

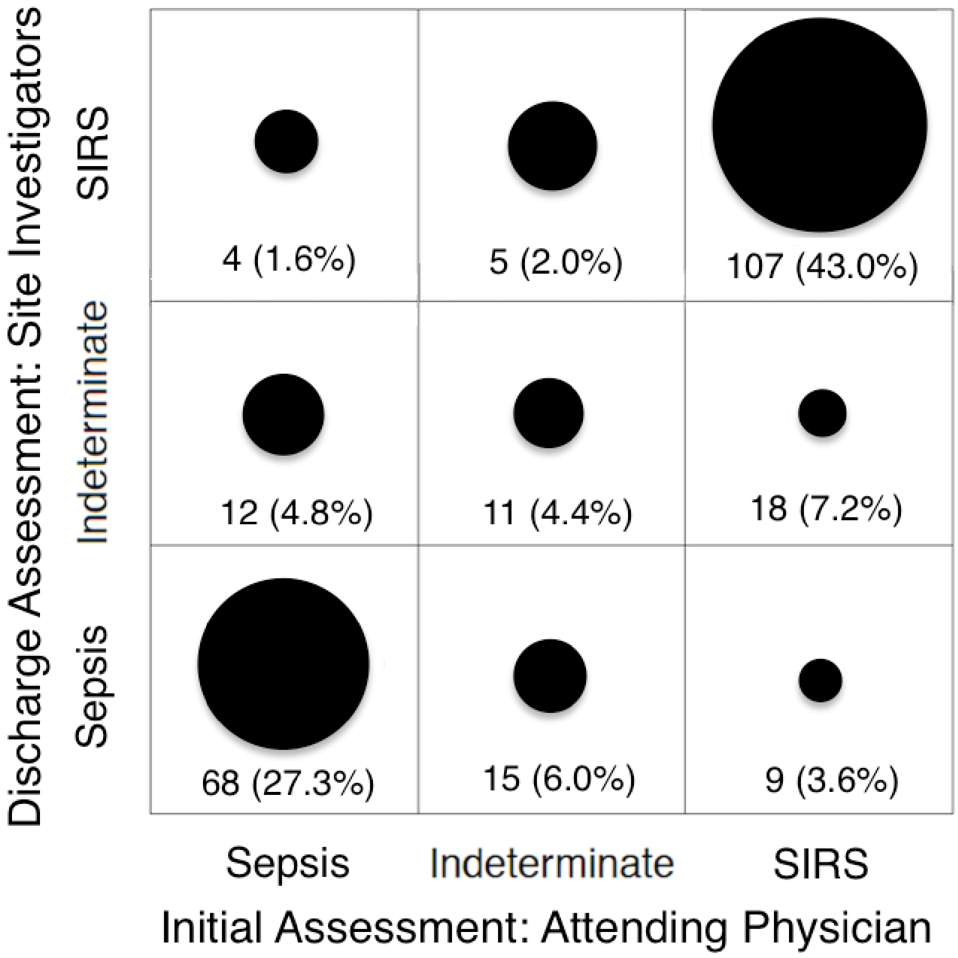

Figure S3-2: Reclassification events between the initial impression by the attending physician, and the RPD.

.

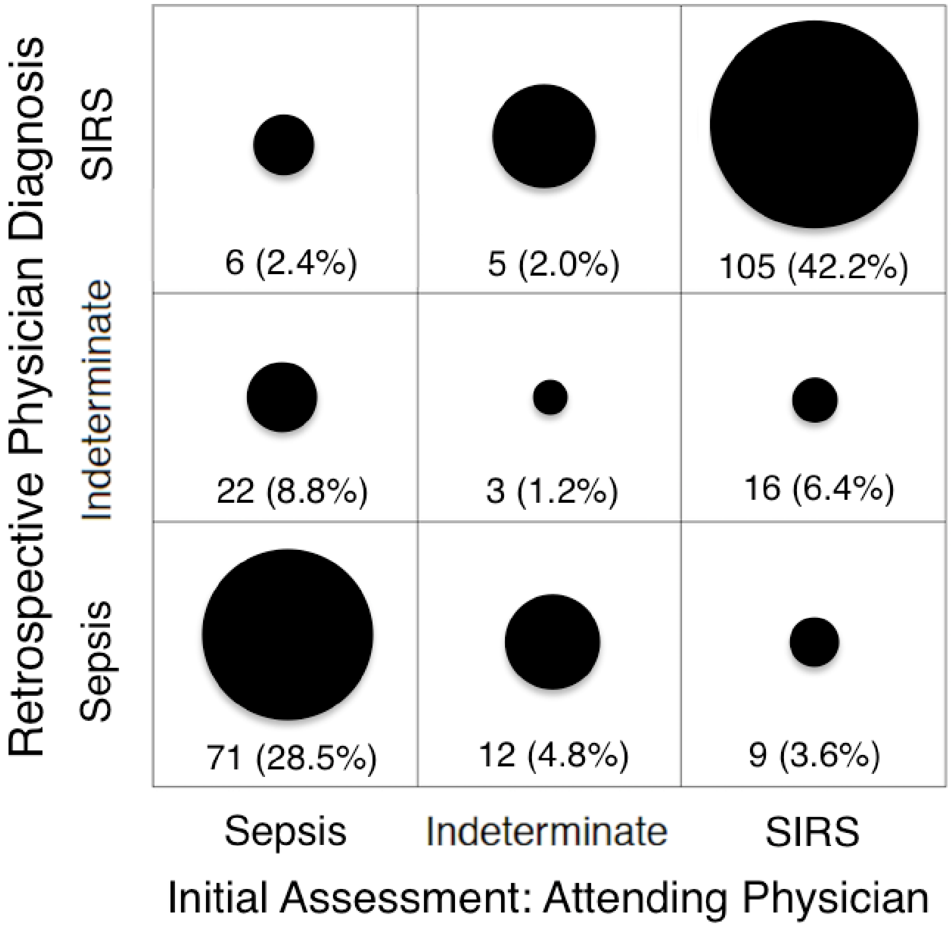

Table S3-1: *Analysis of Reclassification Events*

Reclassification	Number (%) reclassified: attending physician → discharge assessment by site investigators (Fig. S3-1)	Number (%) reclassified: attending physician → RPD (Fig. S3-2)	Change in Apparent Risk Profile	Potential Consequence, Under assumption of an Erroneous Initial Classification
SIRS to sepsis	4 (1.6%)	6 (2.4%)	Low to High	Delayed antibiotic treatment; Prolonged hospital stay; Increased morbidity and mortality
Indeterminate to sepsis	12 (4.8%)	22 (8.8%)	Medium to High	Possible delayed antibiotic treatment
SIRS to Indeterminate	5 (2.0%)	5 (2.0%)	Low to Medium	Possibly delayed treatment
Sepsis to SIRS	9 (3.6%)	9 (3.6%)	High to Low	Excess antibiotic use
Indeterminate to SIRS	18 (7.2%)	16 (6.4%)	Medium to Low	Possible excess antibiotic use
Sepsis to Indeterminate	15 (6.0%)	12 (4.8%)	High to Medium	Possible excess antibiotic use
Total	63 (25.3%)	70 (28.1%)		

Discussion

It is known that early recognition and treatment of sepsis, including treatment with antibiotics, results in better patient outcomes (Rivers, 2010; Liu, 2017). Underuse of antibiotics can put patient lives at risk, while overuse can result in development of resistance and other long-term side effects (Costelloe et al., 2010; Llor & Bjerrum, 2014). Thus, it is important to understand factors that could affect accurate diagnosis of sepsis.

It is reasonable to assume that accuracy of a clinical diagnosis of sepsis will improve as additional clinical data (particularly culture results) become available during transit of a patient through ICU. We found up to ~28% of patients suspected of sepsis were reclassified between the initial diagnosis by the attending physician and the RPD by the external expert panel. Of the reclassified patients, 11.2% were reclassified to sepsis from either SIRS or the indeterminate category. Thus, in the present study, at initial presentation to the attending physician, approximately 1 of 10 patients with an eventual RPD of sepsis were missed.

We assert, therefore, that the observed reclassification rate in this study is a rough estimate of the "correctable error in diagnosis", i.e. error that could potentially be eliminated by the timely and accurate generation of actionable information. Such information could, in theory, be supplied by an appropriate and accurate diagnostic test applied around the time of ICU admission.

There could be a second explanation for the observed reclassification rate: the initial diagnosis using clinical signs at ICU admission might be correct at the time it was made, but the patient's clinical signs may have changed rapidly in the first 24 hours of ICU stay. In our study, the initial assessment by the attending physician was performed using clinical signs at admission, while the discharge assessment by the site investigators and the external RPD were performed using retrospective data

available in the first 24 hours in ICU along with clinical microbiology and other diagnostic test results. This time difference could account for differences in diagnoses.

References

Costelloe C, Metcalfe C, Lovering A, Mant D, Hay AD. Effect of antibiotic prescribing in primary care on antimicrobial resistance in individual patients: systematic review and meta-analysis. BMJ 2010;340:c2096. doi: 10.1136/bmj.c2096. PMID: 20483949

Liu, VX, Fielding-Singh V, Greene JD, Baker JM, Iwashyna TJ, Bhattacharya J, Escobar GJ. The Timing of Early Antibiotics and Hospital Mortality in Sepsis. American Journal of Respiratory and Critical Care Medicine 2017; 196: 856–863. PMID: 28345952

Llor C, Bjerrum L. Antimicrobial resistance: risk associated with antibiotic overuse and initiatives to reduce the problem. Ther Adv Drug Saf. 2014;5(6):229-41. doi: 10.1177/2042098614554919. PMID: 25436105

Rivers, EP. Point: Adherence to Early Goal-Directed Therapy: Does It Really Matter? Yes. After a Decade, the Scientific Proof Speaks for Itself. Chest 2010; 138: 476–480. PMID: 20822986
