# Supplementary material for: Physician agreement on the diagnosis of sepsis in the intensive care unit: estimation of concordance and analysis of underlying factors in a multicenter cohort
Source: J Intensive Care. 2019 Feb 21;7:13. doi: 10.1186/s40560-019-0368-2 (PMC6383290; doi:10.1186/s40560-019-0368-2)
Supplement: Supplementary file 4 — Patients with Respiratory Infections. Figure S4–1. Cumulative distributions of the overall percent agreement statistic for respiratory infections vs. non-respiratory infections + SIRS, calculated from Tables S4–1 and S4–2. The Kolmogorov-Smirnov test indicated a highly significant difference (p < 0.0001). Figure S4–2. Cumulative distributions of the free marginal kappa statistic (κfree) for respiratory infections vs. non-respiratory infections + SIRS, calculated from Tables S4–1 and S4–2. The Kolmogorov-Smirnov test indicated a highly significant difference (p < 0.0001). Figure S4–3. Measured classification discordance in the VENUS + VENUS supplement cohorts (N = 49). (A) Comparison of initial evaluations by attending physician and site investigator (67.3% overall agreement; κfree = 0.51). (B) Comparison of the attending physician’s initial evaluation and site investigators’ discharge evaluation (49.0% overall agreement; κfree = 0.24). (C) Comparison of discharge assessments between site investigators (67.3% overall agreement; κfree = 0.51). (D) Comparison of site investigators’ consensus discharge assessment and external RPD (65.3% overall agreement; κfree = 0.48). Figure S4–4. Measured classification discordance in the VENUS + VENUS supplement cohorts without respiratory infections (N = 207). (A) Comparison of the initial evaluations of the attending physician and the site investigator (80.5% overall agreement; κfree = 0.71). (B) Comparison of attending physician’s initial evaluation and site investigators’ consensus discharge evaluation (81.0% overall agreement; κfree = 0.72). (C) Comparison of discharge assessments between site investigators (93.0% overall agreement; κfree = 0.90). (D) Comparison of site investigators’ consensus discharge assessment and external RPD (91.0% overall agreement; κfree = 0.86). Figure S4–5. Cumulative Distributions of the Indeterminate Vote Fraction, for patients suspected of pneumonia or non-pneumonia respiratory infections (N = 49) [file 40560_2019_368_MOESM4_ESM.pdf]

Physician Agreement on the Diagnosis of Sepsis in the Intensive Care Unit: Estimation of Concordance and Analysis of Underlying Factors in a Multicenter Cohort

Bert K. Lopansri, Russell R. Miller III, John P. Burke, Mitchell Levy, Steven Opal, Richard E. Rothman, Franco R. D'Alessio, Venkataramana K. Sidhaye, Robert Balk, Jared A. Greenberg, Mark Yoder, Gourang Patel, Emily Gilbert, Majid Afshar, Jorge P. Parada, Greg S. Martin, Annette M. Esper, Jordan A. Kempker, Mangala Narasimhan, Adey Tsegaye, Stella Hahn, Paul Mayo, Leo McHugh, Antony Rapisarda, Dayle Sampson, Roslyn A. Brandon, Therese A. Seldon, Thomas D. Yager, Richard B. Brandon

Supplement S4: Patients with Respiratory Infections

Introduction

In this Supplement we analyze the subset of patients diagnosed with respiratory infections (both pneumonia, and non-pneumonia) on the first day of ICU admission. We focus on physicians' attempts to diagnose whether these patients are septic or not. Based on analysis of inter-observer agreement statistics, this diagnosis appears very difficult to make with certainty.

Methods

<u>Inter-observer agreement statistics:</u> We calculated two inter-observer agreement statistics: the % overall agreement, and the free-marginal kappa (κ_{free}). For these calculations, we used the web applet described by Randolph (2005): http://justusrandolph.net/kappa/

A number of different comparisons were performed, as described in the Methods section of the main text: (A) Initial assessment (attending physician) versus initial assessment (site investigator); (B) Initial assessment (attending physician) versus consensus discharge assessment (site investigators); (C) Initial assessment (attending physician) versus external RPD; (D) Initial assessment (site investigator) versus consensus discharge assessment (site investigators); (E) Initial assessment (site investigator) versus external RPD; (F) Consensus discharge assessment (site investigators) versus external RPD; (G,H,I) Comparisons between individual RPD evaluations; (J) Comparisons between the discharge assessments of site investigators.

To obtain an overall sense of the difficulty of diagnosing sepsis in cases of respiratory infection, we performed the following analysis. (1) For the set of patients diagnosed with respiratory infections, we conducted comparisons (A-J) and calculated the cumulative distributions of % overall agreement and free-marginal kappa (κ_{free}). (2) We performed the same operations for the set of patients diagnosed with non-respiratory infections or SIRS. (3) We used the Kolmogorov-Smirnov test to assess the significance of any differences between the cumulative distributions from steps (1) and (2).

<u>Calculation of Indeterminate Vote Fraction:</u> For a given patient, the fraction of votes that were "indeterminate" was calculated as follows: one vote from the attending

physician at admission; one vote from the site investigator at admission; two votes from the two site investigators at discharge; one vote from the adjudicator at discharge (only in cases of disagreement between site investigators); three votes from the three external RPD panelists. The total number of votes therefore was either seven or eight votes, depending on whether or not an adjudicator was used for a particular patient at the discharge evaluation. The fraction of votes that were indeterminate could then be calculated in a straightforward fashion.

Statistical tests: Differences between proportions were evaluated for significance using a two-proportion Z-test (http://www.socscistatistics.com/tests/ztest/Default2.aspx) when sample sizes were large (n*p>5). For small sample sizes (n*p<5) an N-1 chi square test was used instead (https://measuringu.com/ab-cal/). Two-tailed tests were employed. The Kolmogorov-Smirnov test was used to check for significance of differences between cumulative distributions, using an applet available at: www.physics.csbsju.edu/stats/KS-test.html. The significance (p-value) of the Kolmogorov-Smirnov D statistic was crosschecked with an online calculator at http://home.ubalt.edu/ntsbarsh/Business-stat/otherapplets/pvalues.htm#rkstwo.

Results

High frequency of discordant and indeterminate classifications: We examined in greater detail those patients diagnosed with pneumonia (17 in VENUS, 25 in VENUS Supplement, adding up to 42 total). We also performed the analyses including 7 additional patients diagnosed with non-pneumonia respiratory infections (bronchitis, severe influenza, pulmonary edema, tracheitis, lung abscess, pharyngitis, other): 3 from VENUS and 4 from VENUS Supplement. The addition of another 7 patients did not materially alter the conclusions drawn from the 42 pneumonia patients.

An overall summary of findings (% overall agreement and free marginal kappa) is presented in **Table S4-1** for respiratory infections, and in **Table S4-2** for non-respiratory infections + SIRS. **Figure S4-1** compares the cumulative distributions of the free marginal kappa statistic (κ_{free}) for the two conditions. By the Kolmogorov-Smirnov test, the two distributions are very different ($p < 0.0001$).

For all physicians, the patients with respiratory infections (pneumonia and other types) proved to be the most difficult on which to reach consensus, with respect to the presence or absence of sepsis.

***Table S4-1**: Pairwise comparisons: respiratory infections*

Subgroup*	N	Evaluation A	Evaluation B	Overall %	Free Marginal Kappa (κ_{free})
V+Vs pneumonia	42	Admission: Attending physician	Admission: Site investigator	71.4	57.2
V+Vs pneumonia + other respiratory	49	Admission: Attending physician	Admission: Site investigator	67.3	52.0
V+Vs pneumonia	42	Admission: Attending physician	Discharge: Site investigator consensus	50.0	25.0
V+Vs pneumonia + other respiratory	49	Admission: Attending physician	Discharge: Site investigator consensus	49.0	23.5
V+Vs pneumonia	42	Admission: Attending physician	Discharge: Consensus RPD	54.8	32.1
V+Vs pneumonia + other respiratory	49	Admission: Attending physician	Discharge: Consensus RPD	49.0	23.5
V+Vs pneumonia	42	Admission: Site investigator	Discharge: Site investigator consensus	50.0	25.0
V+Vs pneumonia + other respiratory	49	Admission: Site investigator	Discharge: Site investigator consensus	51.0	26.5
V+Vs pneumonia	42	Admission: Site investigator	Discharge: Consensus RPD	59.5	39.3
V+Vs pneumonia + other respiratory	49	Admission: Site investigator	Discharge: Consensus RPD	59.2	38.8
V+Vs pneumonia	42	Discharge: Site investigator A	Discharge: Site investigator B	66.7	50.0
V+Vs pneumonia + other respiratory	49	Discharge: Site investigator A	Discharge: Site investigator B	67.3	51.0
V+Vs pneumonia	42	Discharge: Site investigator consensus	Discharge: Consensus RPD	64.3	46.4
V+Vs pneumonia + other respiratory	49	Discharge: Site investigator consensus	Discharge: Consensus RPD	65.3	48.0
V+Vs pneumonia	42	Discharge: RPD panelist A	Discharge: RPD panelist B	64.3	46.4
V+Vs pneumonia + other respiratory	49	Discharge: RPD panelist A	Discharge: RPD panelist B	65.3	48.0
V+Vs pneumonia	42	Discharge: RPD panelist A	Discharge: RPD panelist C	71.4	57.1
V+Vs pneumonia + other respiratory	49	Discharge: RPD panelist A	Discharge: RPD panelist C	67.3	51.0
V+Vs pneumonia	42	Discharge: RPD panelist B	Discharge: RPD panelist C	71.4	57.1
V+Vs pneumonia + other respiratory	49	Discharge: RPD panelist B	Discharge: RPD panelist C	69.4	54.1

Table S4-2: *Pairwise comparisons: non-respiratory infections + SIRS*

Subgroup	N	Evaluation A	Evaluation B	Overall %	Free Marginal Kappa (κ_{free})
V+Vs non-respiratory + SIRS	200	Admission: Attending physician	Admission: Site investigator	80.5	70.8
V+Vs non-respiratory + SIRS	200	Admission: Attending physician	Discharge: Site investigator consensus	81.0	71.5
V+Vs non-respiratory + SIRS	200	Admission: Attending physician	Discharge: Consensus RPD	77.5	66.2
V+Vs non-respiratory + SIRS	200	Admission: Site investigator	Discharge: Site investigator consensus	85.5	78.2
V+Vs non-respiratory + SIRS	200	Admission: Site investigator	Discharge: Consensus RPD	84.0	76.0
V+Vs non-respiratory + SIRS	200	Discharge: Site investigator A	Discharge: Site investigator B	93.0	89.5
V+Vs non-respiratory + SIRS	200	Discharge: Site investigator consensus	Discharge: Consensus RPD	91.0	86.5
V+Vs non-respiratory + SIRS	200	Discharge: RPD panelist A	Discharge: RPD panelist B	89.5	84.2
V+Vs non-respiratory + SIRS	200	Discharge: RPD panelist A	Discharge: RPD panelist C	83.5	75.2
V+Vs non-respiratory + SIRS	200	Discharge: RPD panelist B	Discharge: RPD panelist C	83.5	75.2

Figure S4-1: *Cumulative distributions of the overall % agreement statistic, for respiratory infections vs. non-respiratory infections + SIRS.* The overall % agreement distributions were calculated from the data reported in Tables S4-1 and S4-2. The Kolmogorov-Smirnov test indicated that the distributions are different, at a high significance level ($p < 0.0001$).

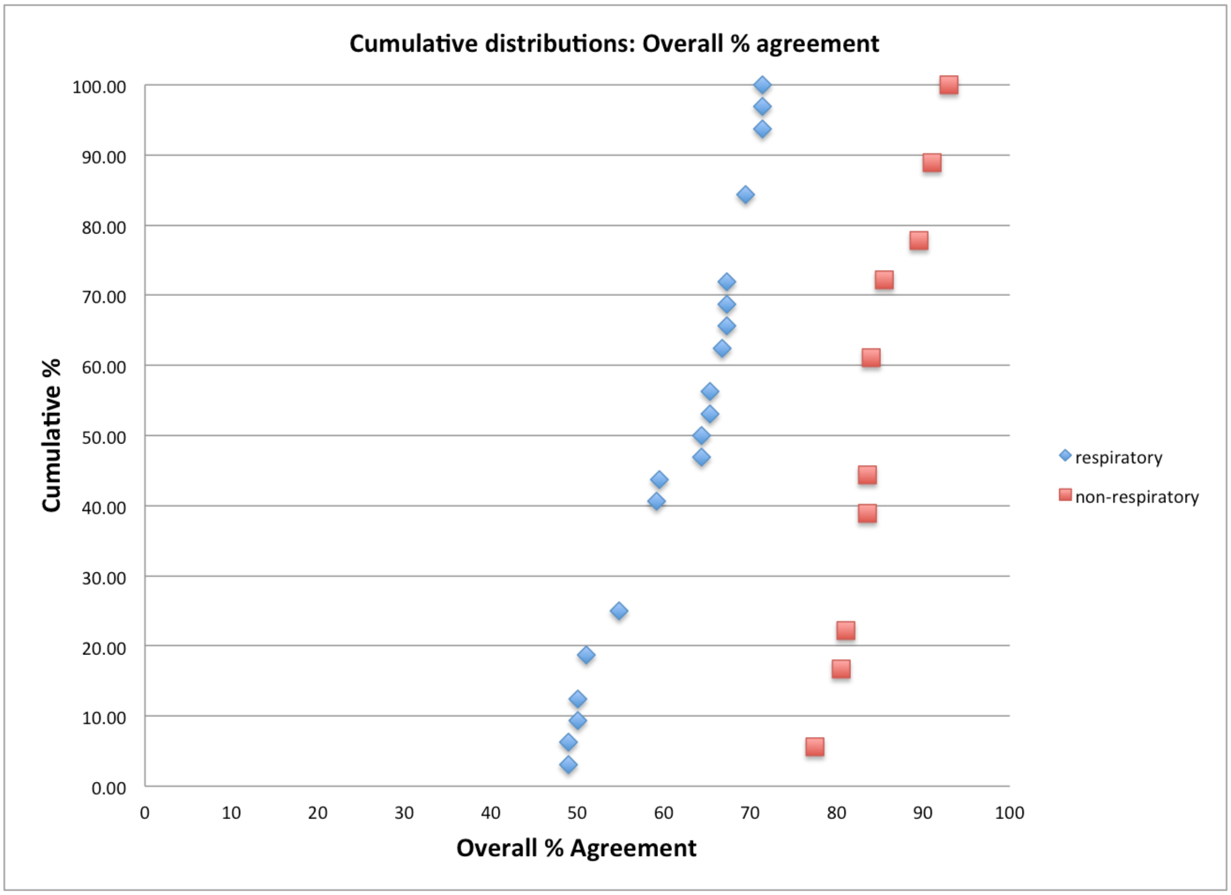

Figure S4-2: *Cumulative distributions of the free marginal kappa statistic (κ_{free}) for respiratory infections vs. non-respiratory infections + SIRS.* Distributions of the free-marginal kappa statistic *(κ_{free})* were calculated from the data reported in Tables S4-1 and S4-2. The Kolmogorov-Smirnov test indicated that the distributions are different, at a high significance level ($p < 0.0001$).

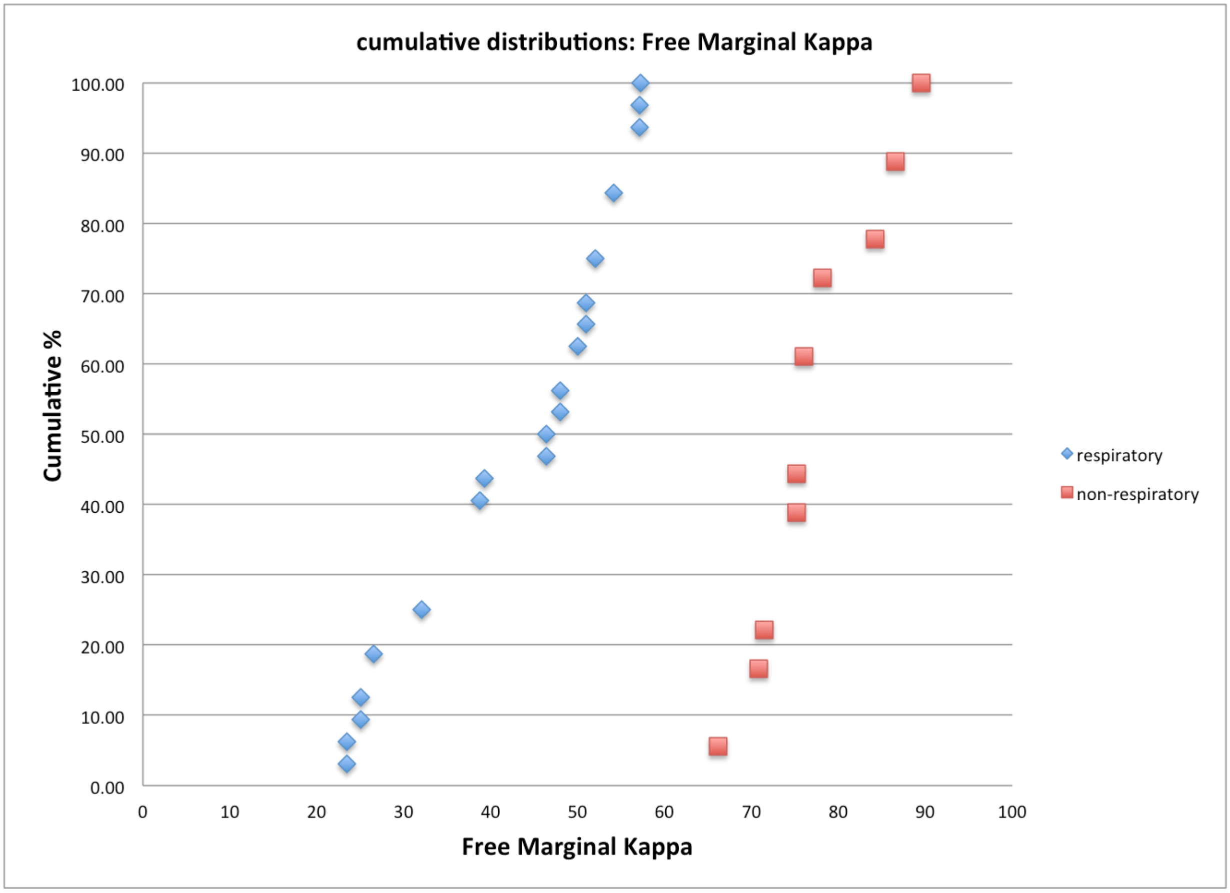

<u>*Respiratory patients classified as SIRS:*</u> In the USA cohort (VENUS + VENUS Supplemental), none of the 49 patients with pneumonia or other respiratory infections were diagnosed initially as SIRS by either the attending physician or the site investigator (**Figure S4-3A**). Upon discharge assessment, 6.1% (3/49) of these patients were diagnosed unanimously as SIRS, by the combination of the site investigators' discharge assessment and the external RPD assessment. (**Figure S4-3D**). This difference in estimated % SIRS between admission and discharge

assessments did not appear statistically significant ($p = 0.08$; chi square test). In sharp contrast to these findings, patients not suspected of pneumonia or other respiratory infections had a significantly ($p < 0.001$) much higher frequency of unanimous SIRS diagnoses, both at ICU admission (108/200, 54.0%; **Figure S4-4A**) and at discharge assessment (122/200, 61.0%; **Figure S4-4D**).

Respiratory patients classified as sepsis: The proportion of patients with pneumonia or other respiratory infections classified as sepsis upon admission by both the attending physician and site investigator (24/49, 49.0%, **Figure S4-3A**) was significantly higher than that of patients not suspected of pneumonia or other respiratory infections (43/200, 21.5%; **Figure S4-4A**) (Z score ≈ 3.89; $p \approx 0.0001$). A smaller (but still significant) difference was observed in the consensus discharge evaluation, by either site investigators or external RPD: 23/49 (46.9%) of patients with pneumonia or other respiratory infections were classified by both as sepsis (**Figure S4-3D**), versus 55/200 (27.5%) of patients with non-respiratory infections or SIRS (**Figure S4-4D**) (Z score ≈ 2.63; $p \approx 0.0085$).

Respiratory patients classified as indeterminate: Finally, the proportion of patients initially classified as indeterminate upon admission by either the attending physican or site investigator was higher for patients with pneumonia or other respiratory infections (24/49 = 49.0%, **Figure S4-3A**) than for patients not suspected of these conditions (38/200 = 19.0%; **Figure S4-4A**). A two-tailed proportions test gave Z-score 4.35 ($p < 0.0001$), indicating a highly significant difference between the two patient groups. If we also considered patients who were indeterminate because of contradictory classifications (sepsis by the attending physician and SIRS by the site investigator, or vice versa), this raised the proportion of Indeterminates in the group not suspected of pneumonia or other respiratory infections to 49/200 = 24.5%, while raising the proportion of indeterminates in the patients not suspected of these conditions to 25/49 = 51.0%. The two-tailed proportions test gave Z score = 3.64 ($p = 0.00028$), still indicating a highly significant difference between groups.

In the site investigators' discharge evaluation, this asymmetry was even greater. The proportion of patients classified as indeterminate by at least one site investigator was 19/49 (38.8%) for patients with pneumonia or other respiratory infections (**Figure S4-3C**) as compared to 27/200 (13.5%) for patients not suspected of these conditions (**Figure S4-4C)**; A two-tailed proportions test gave Z score = 4.09; $p < 0.0001$, indicating a highly significant difference. If we also consider patients who were indeterminate because of contradictory classifications (sepsis by one site investigator and SIRS by the other) this slightly alters the proportions of indeterminates in the patients suspected of pneumonia or other respiratory infections (22/49 = 44.9%) and the remaining patient group (30/200 = 15.0%), which is still a highly significant difference (Z score = 4.61; $p < 0.0001$).

Figure S4-3: *Measured discordance in the classification of SIRS, Indeterminate and sepsis cases, for patients with respiratory infections in the VENUS + VENUS Supplement cohorts (N=49).* (A) Comparison of initial evaluations by attending physician and site investigator (67.3 % overall agreement; κ_{free} = 0.51. (B) Comparison of the attending physician's initial evaluation and the site investigators' discharge evaluation (49.0 % overall agreement; κ_{free} = 0.24). (C) *Comparison of discharge assessments between site investigators* (67.3 % overall agreement; κ_{free} = 0.51). (D) Comparison of site investigators' consensus discharge assessment and external RPD (65.3% overall agreement; κ_{free} = 0.48).

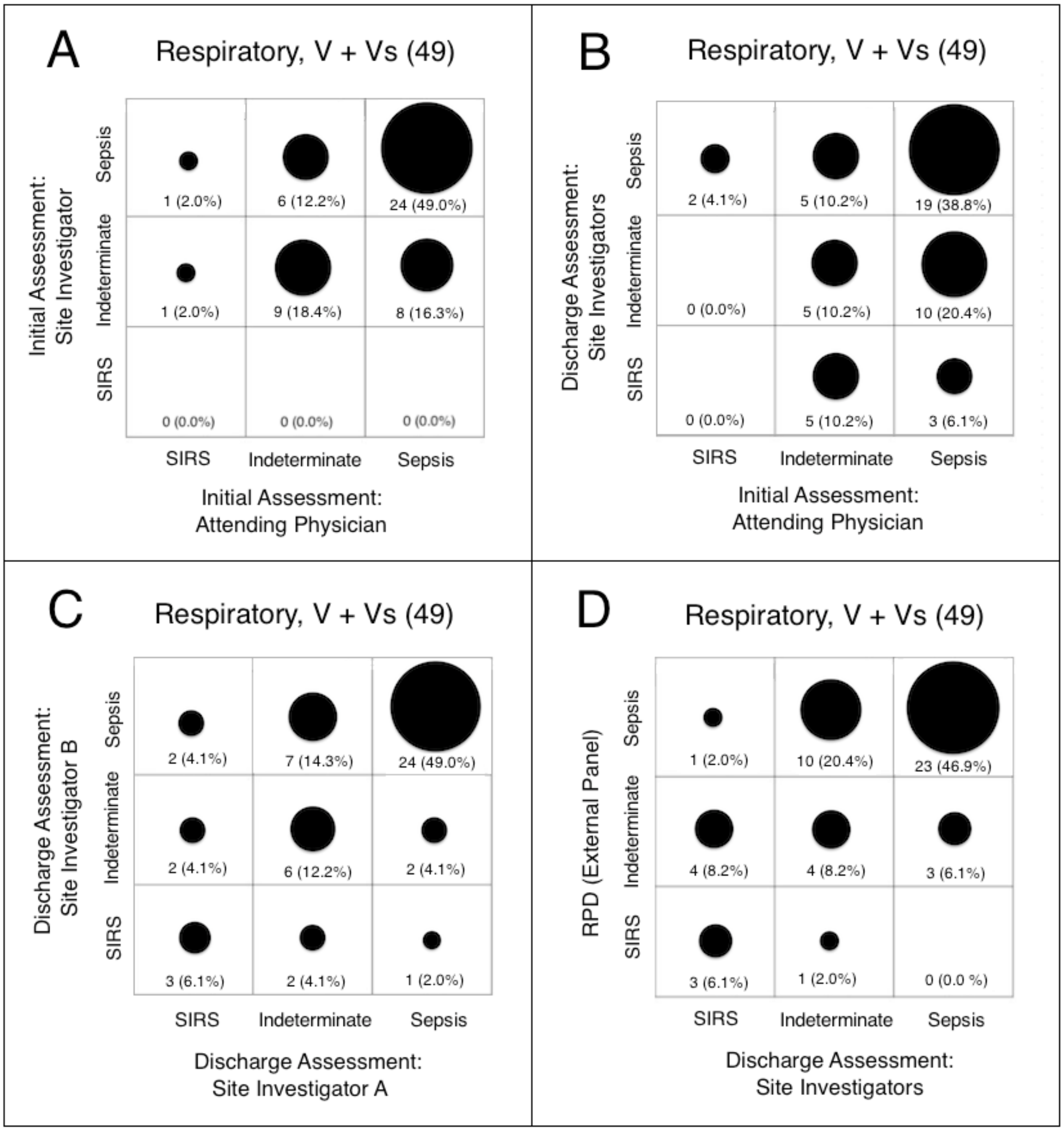

Figure S4-4: *Measured discordance in the classification of SIRS, Indeterminate and sepsis cases, for patients in the VENUS + VENUS Supplement cohorts without respiratory infections (N=207). (A) Comparison of the initial evaluations of the attending physician and the site investigator (80.5% overall agreement;* κ_{free} *= 0.71). (B) Comparison of attending physician's initial evaluation and the site investigators' consensus discharge evaluation (81.0% overall agreement;* κ_{free} *= 0.72). (C) Comparison of discharge assessments between site investigators (93.0 % overall agreement;* κ_{free} *= 0.90). (D) Comparison of site investigators' consensus discharge assessment and external RPD (91.0 % overall agreement;* κ_{free} *= 0.86).*

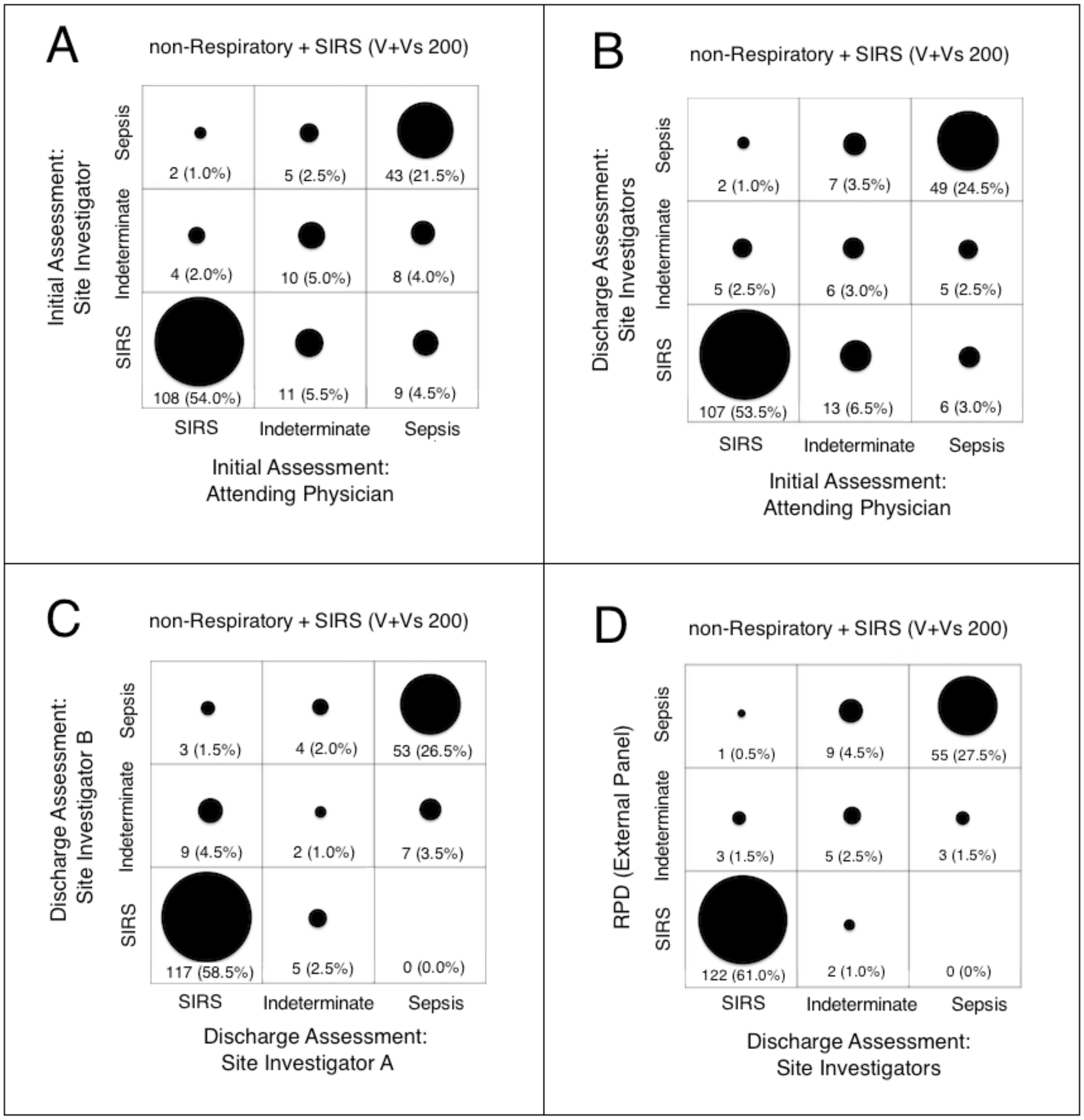

Further analysis of Indeterminates: We performed a further analysis on the indeterminate calls in the patients suspected of pneumonia or non-pneumonia respiratory infections (N=49) versus those with other conditions (N=200). We determined the fraction of all votes that were indeterminate, constructed the cumulative distribution of the indeterminate vote fraction (separately for the two strata), and applied the Kolmogorov-Smirnov (K-S) test to determine the significance of the difference statistic (D) for the two cumulative distributions. The D statistic had the value 0.4064 indicating the two distributions were different at the $p<0.001$ level (**Figure S4-5**). This provides additional evidence to support the claim that the patients with pneumonia or non-pneumonia respiratory infections are especially difficult to diagnose with respect to having sepsis vs. SIRS, as they show a greater fraction of indeterminate calls.

Figure S4-5: *Cumulative Distributions of the Indeterminate Vote Fraction, for the following two strata: patients suspected of pneumonia or non-pneumonia respiratory infections (N=49) versus patients not suspected of these conditions (N=200)*

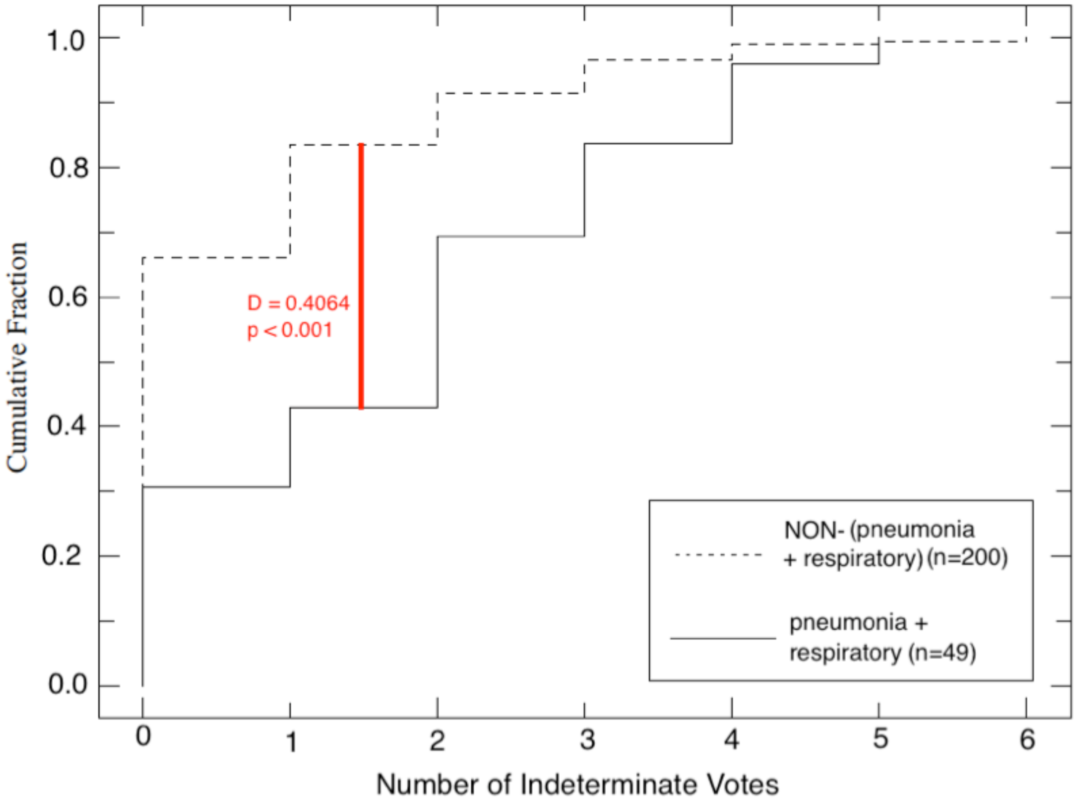

Discussion and Conclusions

We show that physicians display a relatively very low level of agreement, with respect to the diagnosis of sepsis in cases of pneumonia or other respiratory infections. This is observed regardless of which comparison is being made: (1) at admission between attending physician and site investigator; (2) between admission and discharge evaluations; (3) between different discharge evaluations.

References

Randolph, J. J. (2005). Free-marginal multirater kappa: An alternative to Fleiss´ fixed-marginal multirater kappa. Paper presented at the Joensuu University Learning and Instruction Symposium 2005, Joensuu, Finland, October 14-15th, 2005. (ERIC Document Reproduction Service No. ED490661)
