# Supplementary material for: Physician agreement on the diagnosis of sepsis in the intensive care unit: estimation of concordance and analysis of underlying factors in a multicenter cohort
Source: J Intensive Care. 2019 Feb 21;7:13. doi: 10.1186/s40560-019-0368-2 (PMC6383290; doi:10.1186/s40560-019-0368-2)
Supplement: Supplementary file 5 — Stratification by Hospital. Figure S5–1. Plot of overall agreement and κfree for different diagnostic methods at different US hospitals. Values are plotted for (U) the entire USA Cohort (N = 249) and individually for different sub-cohorts: hospital #1 (N = 129), hospital #2 (N = 11), hospital #3 (N = 39), hospital #4 (N = 26), hospital #5 (N = 37), and (6) hospital #6 (N = 7). Note that (U) = hospitals #1 + 2 + 3 + 4 + 5 + 6. Orange bars: overall percent agreement. Blue bars: free-marginal kappa. The comparisons were as follows: (A) initial assessment by attending physician vs. initial assessment by site investigator; (B) initial assessment by attending physician vs. consensus discharge assessment by site investigators; (C) initial assessment by attending physician vs. RPD consensus; (D) initial assessment by site investigator vs. consensus discharge assessment by site investigators; (E) initial assessment by site investigator vs. RPD consensus; (F) consensus discharge assessment by site investigators vs. RPD consensus. Table S5–1. Parameters with significant differences (p < 0.05) between hospital subgroups (hospitals #2, 3) versus (hospitals #5, 6). These two US hospital subgroups displayed the least vs. greatest agreement between the initial diagnosis at admission and later discharge or retrospective diagnoses. (PDF 479 kb) [file 40560_2019_368_MOESM5_ESM.pdf]

Physician Agreement on the Diagnosis of Sepsis in the Intensive Care Unit: Estimation of Concordance and Analysis of Underlying Factors in a Multicenter Cohort

Bert K. Lopansri, Russell R. Miller III, John P. Burke, Mitchell Levy, Steven Opal, Richard E. Rothman, Franco R. D'Alessio, Venkataramana K. Sidhaye, Robert Balk, Jared A. Greenberg, Mark Yoder, Gourang Patel, Emily Gilbert, Majid Afshar, Jorge P. Parada, Greg S. Martin, Annette M. Esper, Jordan A. Kempker, Mangala Narasimhan, Adey Tsegaye, Stella Hahn, Paul Mayo, Leo McHugh, Antony Rapisarda, Dayle Sampson, Roslyn A. Brandon, Therese A. Seldon, Thomas D. Yager, Richard B. Brandon

Supplement S5: Stratification by Hospital

We stratified the USA cohort with respect to different hospital sites, and then evaluated the agreement between different diagnostic methods, for the different hospital sites. This analysis employed the free-marginal kappa statistic (κ_{free}) as appropriate for small sample sizes. We observed significant differences between hospitals with respect to the level of agreement between diagnostic methods (**Figure S5-1**). Specifically, for Hospitals # 2,3 there was relatively high agreement between the initial impressions and the discharge / RPD diagnoses. In contrast, for Hospitals # 5,6 there was relatively low agreement between the initial and later evaluations. The cause(s) of the differences between hospitals identified in Figure S5-1 are not obvious. In comparison to patients from Hospitals # 2,3, the patients from Hospitals # 5,6 could have been more difficult to diagnose, due to differences in

presentation or severity of clinical signs (Table S5-1). Alternatively, the explanation might reside in differences in training or institutional practices.

As a control on the comparison process, the last set of seven columns in Figure S5-1 (comparison F) describes the agreement between the site investigators' discharge evaluations and the external RPD for the entire study cohort (U) and for the individual hospital sites (#1-6). Very high agreement was observed for comparison F, as expected, on the basis of roughly equivalent skill levels of site investigators and RPD panelists, the fact that both had been trained on the study protocol, and also because the RPD panelists had access to the site investigators' discharge evaluations.

We attempted to discern whether any clinical or demographic parameters of the patient groups were the cause(s) of the differences between hospitals identified in **Figure S5-1**. We combined the two US hospital sites with highest inter-observer agreement (Hospitals # 2+3; N=50; κ_{free} 0.78 ± 0.12), and compared these to the combined two hospital sites with lowest inter-observer agreement (Hospitals # 5+6; N=44; κ_{free} 0.43 ± 0.14). Clinical and demographic parameters that were statistically different ($p < 0.05$) between these two subgroups are shown in **Table S5-1**. The analysis revealed that Hospitals # 5+6, with the lower overall agreement between admission and discharge or RPD diagnoses, had a significantly higher number of patients with more SIRS-associated clinical signs (SIRS.N), a higher percentage of black patients, and patients with higher MAP, SeptiScore™ and PCT values, as compared to Hospitals # 2+3. Such parameters might make it more difficult for physicians to agree on a diagnosis in patients suspected of sepsis.

Figure S5-1: *Plot of overall agreement and* κ_{free} *for different diagnostic methods at different US hospitals. Values* are plotted for (U) the entire USA Cohort (N=249), and individually for different sub-cohorts: Hospital #1 (N=129), Hospital #2 (N=11), Hospital #3 (N=39), Hospital #4 (N=26), Hospital #5 (N=37), (6) Hospital #6 (N=7). Note that (U) = Hospitals # 1+2+3+4+5+6. Orange bars: overall % agreement. Blue bars: κ_{free}. The comparisons were as follows: (A) initial assessment by attending physician vs. initial assessment by site investigator; (B) initial assessment by attending physician vs. consensus discharge assessment by site investigators; (C) initial assessment by attending physician vs. RPD consensus; (D) initial assessment by site investigator vs. consensus discharge assessment by site investigators; (E) initial assessment by site investigator vs. RPD consensus; (F) consensus discharge assessment by site investigators vs. RPD consensus.

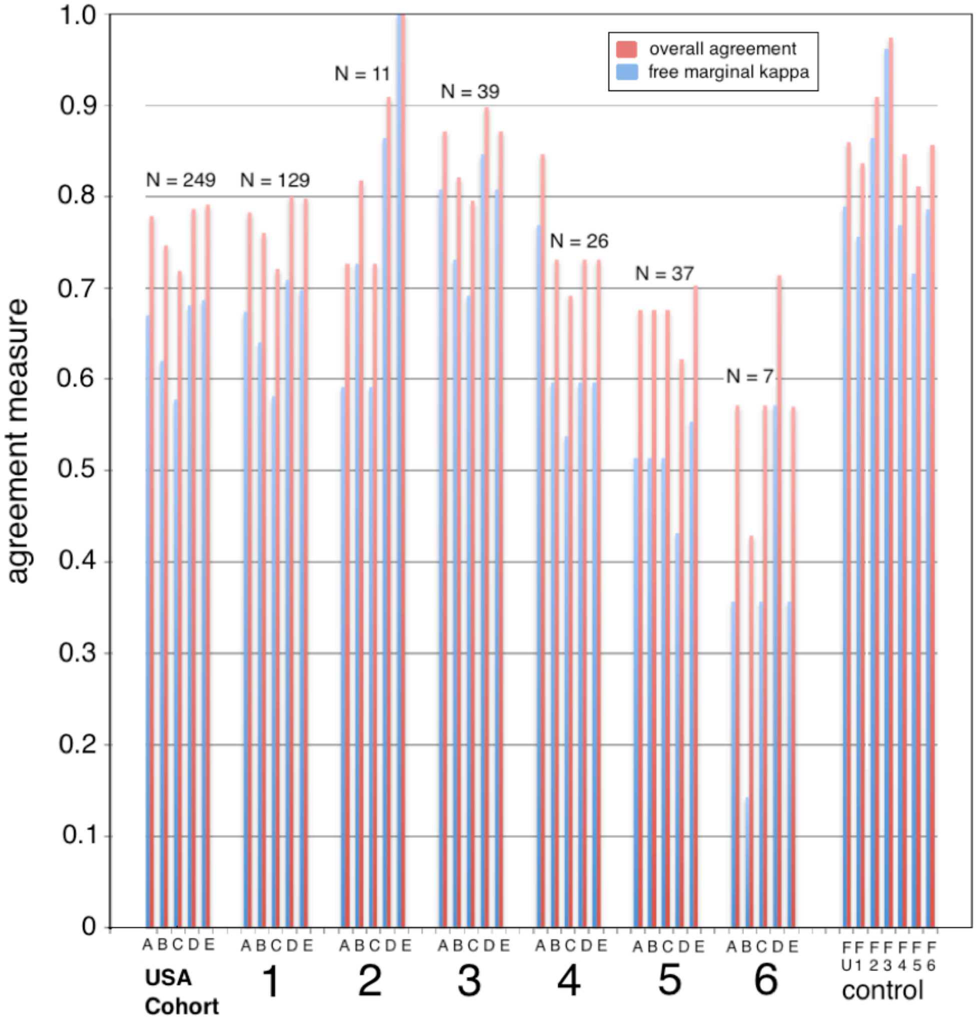

Table S5-1*: Parameters with significant differences ($p < 0.05$) between hospital subgroups (Hospitals # 2,3) and (Hospitals # 5,6).* These two US hospital subgroups displayed the least vs. greatest agreement between the initial diagnosis at admission and later discharge or retrospective diagnoses.

Parameter	Mean ± SD (Hospitals # 2+3)	Mean ± SD (Hospitals # 5+6)	p-value*
Number in group	50	44	
Overall agreement	0.86 ± 0.08	0.62 ± 0.10	0.0032
κ_{free}	0.78 ± 0.12	0.43 ± 0.14	1.2E-04
Race: % Black	23/50 = 46%	30/44 = 68%	0.030
SIRS (N)	2.3 ± 0.5	2.7 ± 0.8	6.2E-03
Highest MAP	96.2 ± 15.8	109.3 ± 21.7	6.7E-03
Lowest MAP	68.9 ± 16.2	78.0 ± 16.7	0.011
Max Temperature	37.2 ± 0.6	37.6 ± 0.9	0.020
SeptiScore	5.0 ± 1.8	5.8 ± 2.2	0.030
Log_2 (PCT)	-2.4 ± 3.7	-0.6 ± 3.9	0.035

* 2-tailed t-test for all parameters except for Overall agreement and Race for which a 2-proportions z-test was used (www.vassarstats.net)
