# Supplementary material for: Physician agreement on the diagnosis of sepsis in the intensive care unit: estimation of concordance and analysis of underlying factors in a multicenter cohort
Source: J Intensive Care. 2019 Feb 21;7:13. doi: 10.1186/s40560-019-0368-2 (PMC6383290; doi:10.1186/s40560-019-0368-2)
Supplement: Supplementary file 6 — Analysis of Indeterminates. Figure S6–1. ROC curve analysis. Panel A: discrimination of sepsis vs. indeterminate, using the variable MAP.Max. Panel B: discrimination of SIRS vs. indeterminate, using the variable Temp.Max. Figure S6–2. Logistic regression model for sepsis vs. indeterminates. The predictor variable is given by the following equation: y = 0.4249 + 0.3672 * SeptiScore + 0.1232 * WBC.Max − 0.0245 * WBC.Min − 0.0269 * MAP.Max. This equation gives AUC = 0.79 (95% CI 0.68–0.90) in ROC curve analysis. Figure S6–3. Logistic regression model for SIRS vs. indeterminates. The predictor variable is given by the following equation: y = 3.1742–0.2548 * log2 PCT − 0.3913 * SeptiScore. This equation gives AUC = 0.81 (95% CI 0.69–0.92) in ROC curve analysis. Table S6–1. Comparison of clinical parameters for patients classified as sepsis, SIRS, or indeterminate, when consensus discharge by site investigators is the comparator. Dataset = Venus + Venus supplement (N = 249). Mean ± SD values are indicated. Significance testing: 2-tailed t test for continuous variables with equal variances assumed (Excel); two-proportion Z-test for categorical variables (http://www.socscistatistics.com/tests/ztest/Default2.aspx). Variables that show significant (p < 0.05) differences between sepsis/indeterminate groups, or between SIRS/indeterminate groups, are highlighted in pink. Table S6–2. Logistic regression to discriminate indeterminates from sepsis. Table S6–3. Logistic regression to discriminate indeterminates from SIRS. Table S6–4. Summary of logistic regression analysis (PDF 558 kb) [file 40560_2019_368_MOESM6_ESM.pdf]

Physician Agreement on the Diagnosis of Sepsis in the Intensive Care Unit: Estimation of Concordance and Analysis of Underlying Factors in a Multicenter Cohort

Bert K. Lopansri, Russell R. Miller III, John P. Burke, Mitchell Levy, Steven Opal, Richard E. Rothman, Franco R. D'Alessio, Venkataramana K. Sidhaye, Robert Balk, Jared A. Greenberg, Mark Yoder, Gourang Patel, Emily Gilbert, Majid Afshar, Jorge P. Parada, Greg S. Martin, Annette M. Esper, Jordan A. Kempker, Mangala Narasimhan, Adey Tsegaye, Stella Hahn, Paul Mayo, Leo McHugh, Antony Rapisarda, Dayle Sampson, Roslyn A. Brandon, Therese A. Seldon, Thomas D. Yager, Richard B. Brandon

Supplement S6: Analysis of Indeterminates

This supplement presents a further analysis of the patients labeled as 'indeterminate' in the consensus discharge evaluation by the site investigators. We attempted to find ways to distinguish the indeteminates from either sepsis or SIRS, based on clinical and laboratory variables.

Definitions

1. A patient was classified as SIRS if both site investigators indicated SIRS;

2. A patient was classified as septic if both site investigators indicated Sepsis;

3. A patient was classified as indeterminate if both site investigators indicated indeterminate.

4. A patient was classified as indeterminate if the two site investigators disagreed with respect to assessment as SIRS, sepsis, or indeterminate, and the third adjudicator did not agree with either site investigator.

Dataset

Table S6-1 summarizes the data (N=249) used in the present analysis. Reference diagnoses were assigned by consensus discharge evaluation. This comparator method led to an assignment of 84 sepsis, 134 SIRS and 31 indeterminates.

We are interested here in the questions of whether, on the basis of clinical data: 1) SIRS can be discriminated from indeterminate, and 2) sepsis can be discriminated from indeterminate.

The analysis proceeded in two steps:

Step 1: clinical variables were identified that showed a significant difference between SIRS vs. indeterminate or sepsis vs. indeterminate, in univariate analysis.

Step 2: an attempt was made to combine the significant univariates by logistic regression, to achieve a better discrimination.

Results

1. Univariate Analysis

Table S6-1 indicates that certain variables (highlighted in pink) showed discrimination between the indeterminate group and either the SIRS group or the sepsis group.

Table S6-1: *Comparison of clinical parameters for patients classified as sepsis, SIRS or Indeterminate, when consensus discharge by site investigators is the comparator.* Dataset = Venus + Venus Supplement (N=249). Mean ± SD values are indicated. Significance testing: t-test for continuous variables, 2-tailed, equal variances assumed (Excel); two-proportion Z-test for categorical variables (http://www.socscistatistics.com/tests/ztest/Default2.aspx). Variables that show significant ($p < 0.05$) differences between sepsis / indeterminate groups, or between SIRS / indeterminate groups, are highlighted in pink.

	Stratum			Significance (Z-score)		
Parameter	Sepsis (N=84)	SIRS (N=134)	Indeterminate (N=31)	Sepsis vs. SIRS	Sepsis vs. Indeterminate	SIRS vs. Indeterminate
Sex: male	45 (54%)	70 (52%)	17 (55%)	0.85 (Z=0.19)	0.90 (Z=0.12)	0.80 (Z=0.26)
Race: white	52 (62%)	91 (68%)	19 (61%)	0.36 (Z=0.91)	0.95 (Z=0.06)	0.48 (Z=0.70)
Age	57.8 ± 15.8	55.0 ± 17.0	61.6 ± 17.8	0.22	0.26	5.3E-02
ICU LoS	4.8 ± 5.9	2.4 ± 3.2	6.4 ± 9.1	2.6E-04	0.25	6.1E-05
Hospital LoS	11.2 ± 12.8	5.8 ± 4.4	8.9 ± 8.7	9.8E-06	0.34	5.0E-03
Ventilator	13 (15%)	23 (17%)	8 (26%)	0.74 (Z=0.33)	0.20 (Z=1.27)	0.27 (Z=1.11)
Pneumonia	20 (24%)	7 (5%)	15 (48%)	<1E-04 (Z=4.05)	1.1E-02 (Z=2.54)	<1E-04 (Z=6.37)
Pos. viral tests	8 (10%)	8 (6%)	4 (13%)	0.33 (Z=0.98)	0.60 (Z=0.53)	0.18 (Z=1.34)
# SIRS criteria	3.1 ± 0.8	2.6 ± 0.7	3.0 ± 0.8	9.4E-06	0.68	5.5E-03
APACHE score	76 ± 37	61 ± 35	73 ± 42	2.0E-03	0.63	0.11
SOFA score	6.4 ± 4.1	5.0 ± 2.9	6.2 ± 3.4	1.0E-02	0.87	4.5E-02
WBC.Max	16.9 ± 9.0	12.8 ± 6.3	11.9 ± 6.3	1.0E-04	5.2E-03	0.48
WBC.Min	12.9 ± 7.3	9.8 ± 4.6	9.4 ± 5.8	1.7E-04	1.8E-02	0.68
Glucose.Max	182 ± 111	202 ± 156	172 ± 132	0.32	0.68	0.32
Glucose.Min	128 ± 46	132 ± 63	122 ± 48	0.66	0.53	0.42
MAP.max	105 ± 25	114 ± 23	122 ± 29	1.7E-02	6.5E-03	0.13
MAP.min	57 ± 15	65 ± 18	63 ± 17	4.1E-04	8.2E-02	0.47
Temp.Max	38.2 ± 1.0	37.4 ± 0.7	37.9 ± 0.8	2.0E-10	0.18	4.7E-04
Temp.Min	36.2 ± 0.9	36.1 ± 0.7	36.1 ± 0.7	0.29	0.87	0.55
HeartRate.Max	120 ± 25	113 ± 25	122 ± 26	3.4E-02	0.68	5.8E-02
HeartRate.Min	79 ± 20	74 ± 16	76 ± 16	6.8E-02	0.47	0.61
PCT	22.7 ± 40.6	2.2 ± 9.6	14.9 ± 32.2	9.0E-06	0.44	1.9E-03
$\log_2$(PCT)	1.9 ± 3.5	-2.8 ± 3.0	0.6 ± 3.6	3.5E-15	0.15	3.2E-05
SeptiScore	6.8 ± 2.0	4.0 ± 1.4	5.2 ± 1.7	4.9E-25	1.4E-04	1.5E-04
SeptiScore Band	3.4 ± 0.9	2.2 ± 0.9	2.9 ± 1.1	2.2E-19	5.8E-03	3.8E-04

Based on some of the variables in Table S6-1, the indeterminates appear more like SIRS, while based on other variables they appear more like sepsis. This may help to explain why the patients are classified as indeterminate in the first place. Consider, for example, the two panels of **Figure S6-1**:

***Panel A:** Sepsis vs. Indeterminate, Clinical Variable = MAP.Max*

Comparison of the sepsis group (N=64) and the indeterminate group (N=23)
(20 sepsis patients and 8 indeterminates not used, because MAP.MAX data missing).
The two groups are significantly different, by t-test (p = 6.5E-03).
A ROC analysis of the two groups gives AUC = 0.70 (95% CI: 0.57-0.82).
Thus, according to MAP.Max, the indeterminates are more like SIRS patients.

***Panel B:** SIRS vs. Indeterminate, Clinical Variable = Temp.Max*

Comparison of the SIRS group (N=134) and the indeterminate group (N=31).
The two groups are significantly different, by t-test (p = 4.7E-04).
A ROC analysis of the two groups gives AUC = 0.68 (95% CI: 0.57-0.78).
Thus, according to Temp.Max the indeterminates are more like sepsis patients.

***Figure S6-1:** ROC Curve analysis. Panel A: Discrimination of sepsis vs. indeterminate, using the variable MAP.Max. Panel B: Discrimination of SIRS vs. indeterminate, using the variable Temp.Max.*

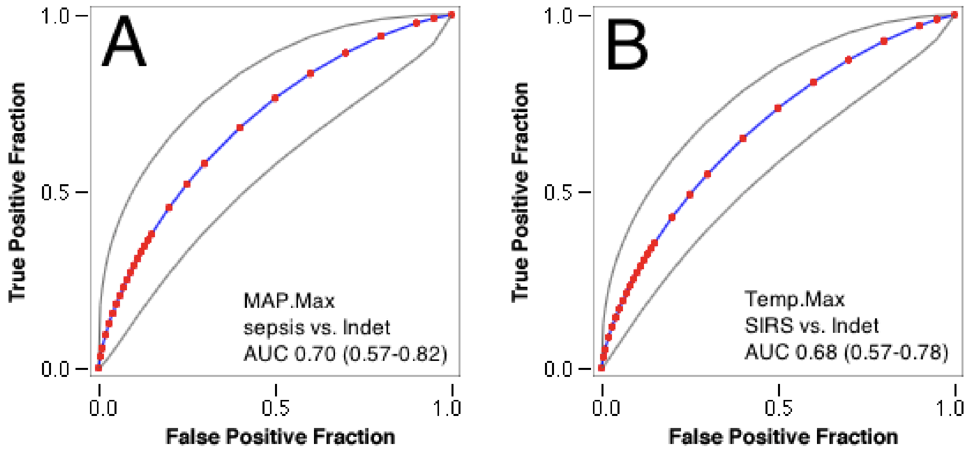

2. Logistic Regresssion Analysis: Sepsis vs. Indeterminate

A logistic regression model was created, to find a classifier for separating septic patients from indeterminates, based on clinical signs and/or molecular biomarkers. The model used the consensus discharge diagnosis as the comparator, and analyzed 23 indeterminates and 64 septic patients from the Venus + Venus Supplemental cohorts. We excluded 8 indeterminates and 20 sepsis patients

because of missing values. An online logistic regression calculator (http://statpages.info/logistic.html) was used.

Overall Model Fit, as indicated in **Table S6-2**: Chi Square = 20.6867; df=4; p = 0.0004. The significance of the variables decreased in the order SeptiScore > WBC.Max ≈ WBC.Min ≈ MAP.Max, based on the magnitude of the Odds Ratio.

Table S6-2*: Logistic regression to discriminate Indeterminates from Sepsis*

Variable	Coeff.	Std Err	p	Odds Ratio	95% CI LB	95% CI UB
MAP.Max	-0.0269	0.0107	0.0118	0.9734	0.9532	0.9941
WBC.Max	0.1232	0.0892	0.1674	1.1311	0.9496	1.3471
WBC.Min	-0.0245	0.1005	0.8071	0.9757	0.8013	1.1882
SeptiScore	0.3672	0.1524	0.0160	1.4437	1.0708	1.9464
Intercept	0.4249	1.5318	0.7815			

The resultant logistic regression model was then used in ROC curve analysis (http://www.rad.jhmi.edu/jeng/javarad/roc/JROCFITi.html). This analysis produced the result AUC = 0.79 (95% CI: 0.68-0.90), as indicated in **Figure S6-2**.

Figure S6-2: *Logistic regression model for sepsis vs. Indeterminates.* The predictor variable is given by the following equation: y = 0.4249 + 0.3672 * SeptiScore + 0.1232 * WBC.Max - 0.0245 * WBC.Min - 0.0269 * MAP.Max. This equation gives AUC = 0.79 (95% CI: 0.68-0.90) in ROC curve analysis.

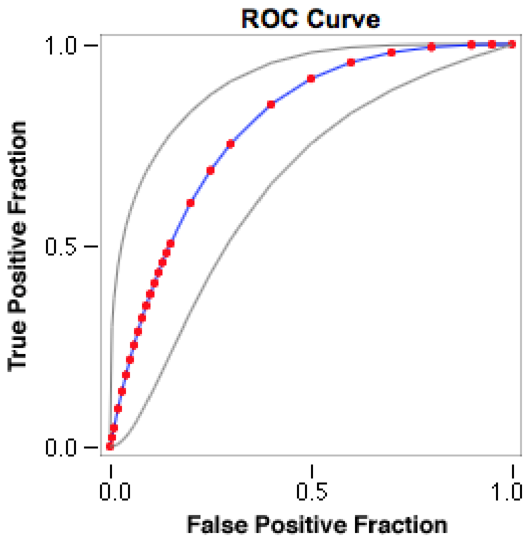

3. Logistic Regression Analysis: SIRS vs. Indeterminate

A complementary logistic regression analysis was conducted, to discriminate SIRS patients from indeterminates. Table S5-1 identified eight variables with significant univariate differences between SIRS and indeterminate categories (age, ICU LoS, hospital LoS, N.SIRS, SOFA, Temp.Max, log2 PCT, SeptiScore). However, we chose to construct the logistic model with only two variables (log2 PCT, SeptiScore) to avoide the possibility of overfitting. The model used consensus discharge diagnosis as the comparator, and analyzed 15 indeterminates and 73 SIRS patients from the Venus + Venus Supplemental cohorts. We excluded 6 intermediates and 29 SIRS patients because of missing values. As before, an online logistic regression calculator (http://statpages.info/logistic.html) was used.

Overall Model Fit, as indicated in **Table S6-3**: Chi Square = 18.3862; df=2; p = 0.0001. SeptiScore and log2 PCT are both highly significant in this logistic model. ROC curve analysis gave the result AUC = 0.81 (95% CI: 0.69-0.92) as indicated in **Figure S6-3**.

***Table S6-3**: Logistic regression to discriminate Indeterminates from SIRS*

Variable	Coeff.	Std Err	p	Odds Ratio	95% CI LB	95% CI UB
log2 PCT	-0.2548	0.0940	0.0067	0.7751	0.6446	0.9319
SeptiScore	-0.3913	0.2215	0.0772	0.6762	0.4381	1.0437
Intercept	3.1742	1.1803	0.0072			

Figure S6-3: *Logistic regression model for SIRS vs. Indeterminates.* The predictor variable is given by the following equation: y = 3.1742 - 0.2548 * log2 PCT - 0.3913 * SeptiScore. This equation gives AUC = 0.81 (95% CI: 0.69-0.92) in ROC curve analysis.

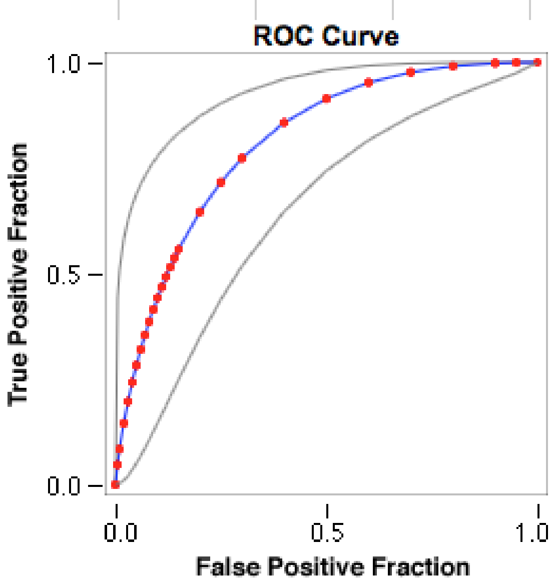

These two regression analyses indicate that the Indeterminate group can be resolved from either the Sepsis group or the SIRS group, using different sets of clinical variables in each case, as summarized in **Table S6-4**:

Table S6-4: *Summary of Logistic Regression Analysis*

Discrimination	Equation	AUC (95% CI)
Sepsis vs. Indeterminate	y = 0.4249 + 0.3672 * SeptiScore + 0.1232 * WBC.Max - 0.0245 * WBC.Min - 0.0269 * MAP.Max	0.79 (95% CI: 0.68-0.90)
SIRS vs. Indeterminate	y = 3.1742 - 0.2548 * log2 PCT - 0.3913 * SeptiScore	0.81 (95% CI: 0.69-0.92)
