# Supplementary material for: Physician agreement on the diagnosis of sepsis in the intensive care unit: estimation of concordance and analysis of underlying factors in a multicenter cohort
Source: J Intensive Care. 2019 Feb 21;7:13. doi: 10.1186/s40560-019-0368-2 (PMC6383290; doi:10.1186/s40560-019-0368-2)
Supplement: Supplementary file 7 — Analysis of Treated vs. Untreated SIRS Patients. Figure S7–1. Behavior of logistic regression models in ROC curve analysis. (A) Five variable model from Table S7–2, giving AUC = 0.72 (95% CI 0.63–0.81). (B) Four variable model from Table S7–3, giving AUC = 0.71 (95% CI 0.62–0.80). Figure S7–2. Machine learning attempts to identify combinations of clinical variables and demographic variables that discriminate between antibiotic treatment and no treatment, within the SIRS group. Recursive feature elimination was used, within a logistic regression (LR) or Random Forests (RF) model. Figure S7–3. Gini ranking of individual words in the “physician comments” field of the case report form, for SIRS patients. The ranking is based on contribution toward discriminating antibiotic-treated vs. untreated SIRS patients. Abbreviations: cxr, chest x-ray; dka, diabetic ketoacidosis; mri, magnetic resonance imaging. Figure S7–4. Gini ranking of word-pairs in the “physician comments” field of the case report form, for SIRS patients. The ranking is based on contribution toward discriminating antibiotic-treated vs. untreated SIRS patients. Abbreviation: chf, congestive heart failure. Figure S7–5. Machine learning attempt to identify combinations of clinical variables, demographic variables, words, and word-pairs that discriminate between antibiotic treatment and no treatment, within the SIRS group. Recursive feature elimination was used, within a logistic regression (LR) or Random Forests (RF) model. Table S7–1. Test for ability of clinical and demographic parameters to distinguish between SIRS patients who received (AB+) or did not receive (AB−) therapeutic antibiotics. Diagnosis was by consensus RPD. Parameters are listed in order of decreasing significance (2-tailed p-value) as evaluated either by Student’s t test, assuming equal variances in the two groups (for continuous variables), or by a 2-proportions z-test (www.vassarstats.net) for categorical variables. Table S7–2. Use of logi [file 40560_2019_368_MOESM7_ESM.pdf]

Physician Agreement on the Diagnosis of Sepsis in the Intensive Care Unit: Estimation of Concordance and Analysis of Underlying Factors in a Multicenter Cohort

Bert K. Lopansri, Russell R. Miller III, John P. Burke, Mitchell Levy, Steven Opal, Richard E. Rothman, Franco R. D'Alessio, Venkataramana K. Sidhaye, Robert Balk, Jared A. Greenberg, Mark Yoder, Gourang Patel, Emily Gilbert, Majid Afshar, Jorge P. Parada, Greg S. Martin, Annette M. Esper, Jordan A. Kempker, Mangala Narasimhan, Adey Tsegaye, Stella Hahn, Paul Mayo, Leo McHugh, Antony Rapisarda, Dayle Sampson, Roslyn A. Brandon, Therese A. Seldon, Thomas D. Yager, Richard B. Brandon

Supplement S7: Analysis of treated vs. untreated SIRS patients

This supplement presents a detailed analysis of patients that were classified as SIRS and either treated or not treated with therapeutic antibiotics. It is possible that clinicians intuitively used some combination of clinical and demographic parameters as the basis of this decision. It is also possible that certain discriminating patient characteristics might have been captured as text in the 'physician comments' field of the case report forms.

We asked if the decision to treat a SIRS patient, or to leave this patient untreated, was correlated with any combination of clinical, demographic or textual variables:

> *Ho (null hypothesis):* for SIRS patients, the decision to treat or not is not correlated with any detectable combination of clinical, demographic and textual variables.

Methods

1. Univariate Analysis

Consensus RPD by the external panel was used as the comparator to identify SIRS patients. Individual clinical and demographic variables were tested for the ability to discriminate treated vs. untreated SIRS patients, either with Student's t-test (Microsoft Excel; continuous variables) or by a 2-proportions Z-test (www.vassarstats.net; categorical variables).

2. Logistic Regression

The five most significant variables from the univariate analysis ($p < 0.022$) were then combined in logistic regression, to investigate if this combination could lead to improved discrimination of treated vs. untreated SIRS patients. The following logistic regression applet was used: http://statpages.info/logistic.html

3. Machine Learning

We also analyzed the SIRS patient subgroup (106/249; 42.6% of total, as defined by unanimous agreement) by machine learning methods, in an attempt to identify factors that could explain the treat / no treat decision. Two approaches were employed. (1) We used recursive feature elimination (Guyon et al. 2002; Kuhn 2018) with either logistic regression or Random Forests (Brieman 2001; Liaw 2012), to search for informative classifiers that were combinations of the following variables: N.SIRS, ICU.LoS, Hospital.LOS, Age, HeartRate.Max, HeartRate.Min, APACHE.Score, Mean.Art.Pressure.Min, WBC.Max, WBC.Min, Glucose.Max, Glucose.Min. We also repeated the exercise, adding in the following binary (+/-) variables: culture.blood, culture.drain, culture.pus, culture.respiratory, culture.skin, culture.sputum, culture.urine, culture.other.contaminates. (2) We performed a linguistic analysis of single words and word pairs in the 'physician comments' field of the patient case reports, using a Random Forests approach. (3) We pooled the most informative clinical and demographic parameters, words and word-pairs from above, and then repeated the recursive variable elimination process with either logistic regression or Random Forests.

Results

1. Univariate Analysis

Table S7-1 presents a list of clinical and demographic parameters that were analyzed, in order of decreasing statistical significance, for the discrimination of treated vs. untreated SIRS patients.

Table S7-1: *Test for ability of clinical and demographic parameters to distinguish between SIRS patients who received (AB+) or did not receive (AB-) therapeutic antibiotics.* Diagnosis of the SIRS subjects (N=130 total) was by consensus RPD. Parameters are listed in order of decreasing significance (2-tailed p-value) as evaluated either by Student's t-test, assuming equal variances in the two groups (for continuous variables), or by a 2-proportions z-test (www.vassarstats.net) for categorical variables.

**Parameter**[1]	**Number of data points avaliable**[2]		**p-value**
	**Patients receiving antibiotics**	**Patients not receiving antibiotics**	
MAP.Min	75	52	0.003
HR.Max	78	52	0.007
Temp.Max	78	52	0.011
Hospital LoS	78	52	0.014
N.SIRS	78	52	0.022
SOFA	54	36	0.043
$\log_2$ PCT	51	34	0.054
Apache	78	51	0.080
WBC.Min	78	51	0.153
pH	34	17	0.183
Age	78	52	0.272
race: non-white	23/78 (29.5%)	20/52 (38.5%)	0.286
race: white	55/78 (70.5%)	32/52 (61.5%)	0.286
WBC.Max	78	51	0.352
Glucose.Max	68	46	0.406
SeptiScore	78	52	0.413
sex: female	35/78 (44.9%)	27/52 (51.9%)	0.430
sex: male	43/78 (55.1%)	25/52 (48.1%)	0.430
Lactate	39	17	0.473

Parameter[1]	Number of data points avaliable[2]		p-value
	Patients receiving antibiotics	Patients not receiving antibiotics	
MAP.Max	58	42	0.694
ICU LoS	78	52	0.749
Temp.Min	74	51	0.760
HR.Min	78	52	0.960

[1]Abbreviations: Glucose.Max, maximum blood glucose concentration; HR.Max, maximum heart rate; HR.Min, minimum heart rate; ICU LoS, length of stay in ICU (days); MAP.Max, maximum mean arterial blood pressure; MAP.Min, minimum mean arterial blood pressure; N.SIRS, number of SIRS criteria met; Temp.Max, maximum core temperature; Temp.Min, minimum core temperature; WBC.Max, maximum white blood cell count; WBC.Min, minimum white blood cell count.

[2]No imputation of missing values was performed.

2. Logistic Regression

The results of a logistic regression analysis employing the five most significant variables from Table S7-1 are shown in **Table S7-2**. We also conducted the analysis after removing Hospital.LoS (hospital length of stay) as a variable, as it could not play a role in diagnosis within the first 24 hours of ICU admission. Results are given in **Table S7-3** (four variable model). There is no significant difference in AUC between the two models, as shown in the ROC plots of **Figure S7-1**.

Table S7-2: *Use of logistic regression, to discriminate between SIRS patients who were treated vs. not treated with antibiotics.* Five variable model.

Parameter	coeff	SE	p	OR	95% LB	95% UB
MAP.Min	-0.0200	0.0128	0.1180	0.9802	0.9560	1.0051
HR.Max	0.0128	0.0097	0.1874	1.0129	0.9938	1.0324
Temp.Max	0.4540	0.3611	0.2087	1.5745	0.7759	3.1953
Hospital LoS	0.0906	0.0546	0.0970	1.0949	0.9837	1.2186
N.SIRS	0.2472	0.3117	0.4278	1.2805	0.6950	2.3590
Intercept	-17.8210	13.3024	0.1803			

***Table S7-3:** Use of logistic regression, to discriminate between SIRS patients who were treated vs. not treated with antibiotics.* Four variable model.

Parameter	coeff	SE	p	OR	95% LB	95% UB
MAP.Min	-0.0239	0.0128	0.0616	0.9764	0.9523	1.0012
HR.Max	0.0125	0.0096	0.1920	1.0125	0.9938	1.0317
Temp.Max	0.4372	0.3602	0.2249	1.5484	0.7643	3.1370
N.SIRS	0.2386	0.3036	0.3502	1.3280	0.7324	2.4077
Intercept	-16.5106	13.2746	0.2136			

***Figure S7-1:** Behavior of logistic regression models in ROC curve analysis.* **(A)** Five variable model from Table S7-2, giving AUC = 0.72 (95% CI: 0.63-0.81). **(B)** Four variable model from Table S7-3, giving AUC = 0.71 (95% CI: 0.62-0.80).

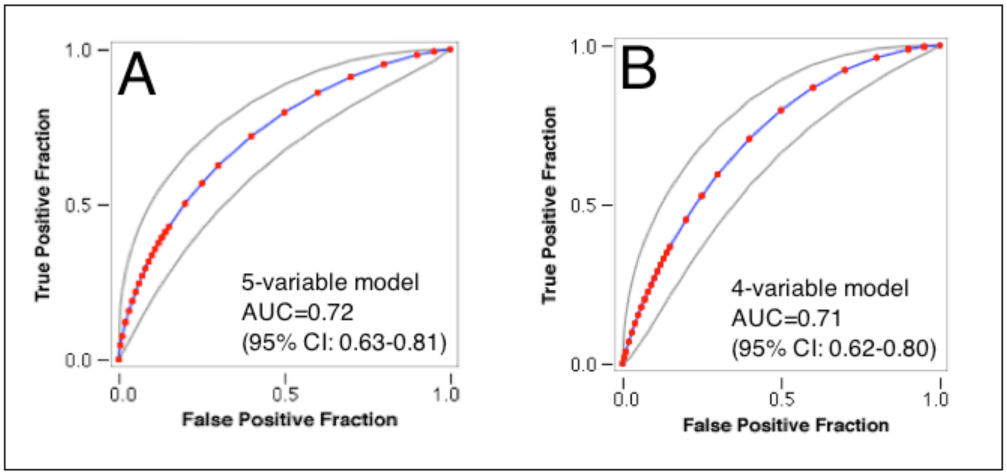

3. Machine Learning

To analyze the treat / no treat decision from a machine-learning perspective, we first used recursive feature elimination within a logistic regression or Random Forests model. The feature elimination process started with twelve multi-valued parameters (N.SIRS, ICU.LoS, Hospital.LOS, Age, HeartRate.Max, HeartRate.Min, APACHE.Score, Mean.Art.Pressure.Min, WBC.Max, WBC.Min, Glucose.Max, Glucose.Min) and eight binary-valued (+/-) parameters (culture.blood, culture.drain, culture.pus, culture.respiratory, culture.skin, culture.sputum, culture.urine, culture.other.contaminates). The feature-elimination trajectories from this approach are shown in **Figure S7-2**.

Figure S7-2: *Machine learning attempt to identify combinations of clinical variables and demographic variables that discriminate between antibiotic treatment and no treatment, within the SIRS group. Recursive feature elimination was used, within a logistic regression (LR) or Random Forests (RF) model.*

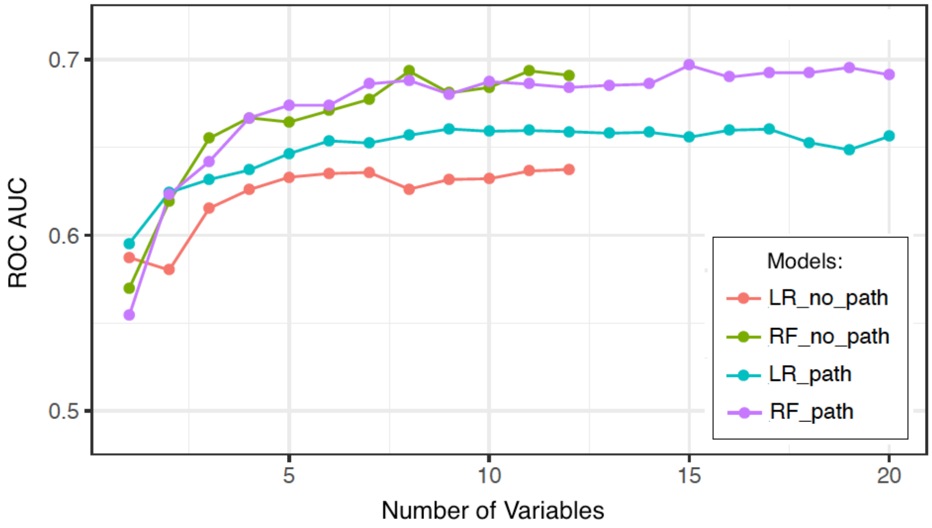

With an asymptotic value AUC → 0.7, we found these recursive feature elimination approaches to perform no better than our original logistic regression analysis (Figure S7-1).

We next turned to a linguistic analysis of the 'physician comments' field in the patient case reports. After removing all non-informative words ("or", "if", "but", "on", etc.), the words that remained were used as binary classifier variables in a Random Forest analysis. The Gini Index was used to rank the words in order of increasing importance (**Figure S7-3**), and the resultant ROC analysis gave AUC = 0.63 ± 0.05.

Figure S7-3: *Gini ranking of individual words in the 'physician comments' field of the case report form, for SIRS patients.* The ranking is based on contribution toward discriminating antibiotic-treated vs. untreated SIRS patients. Abbreviations: cxr, chest X-ray; dka, diabetic ketoacidosis; mri, magnetic resonance imaging.

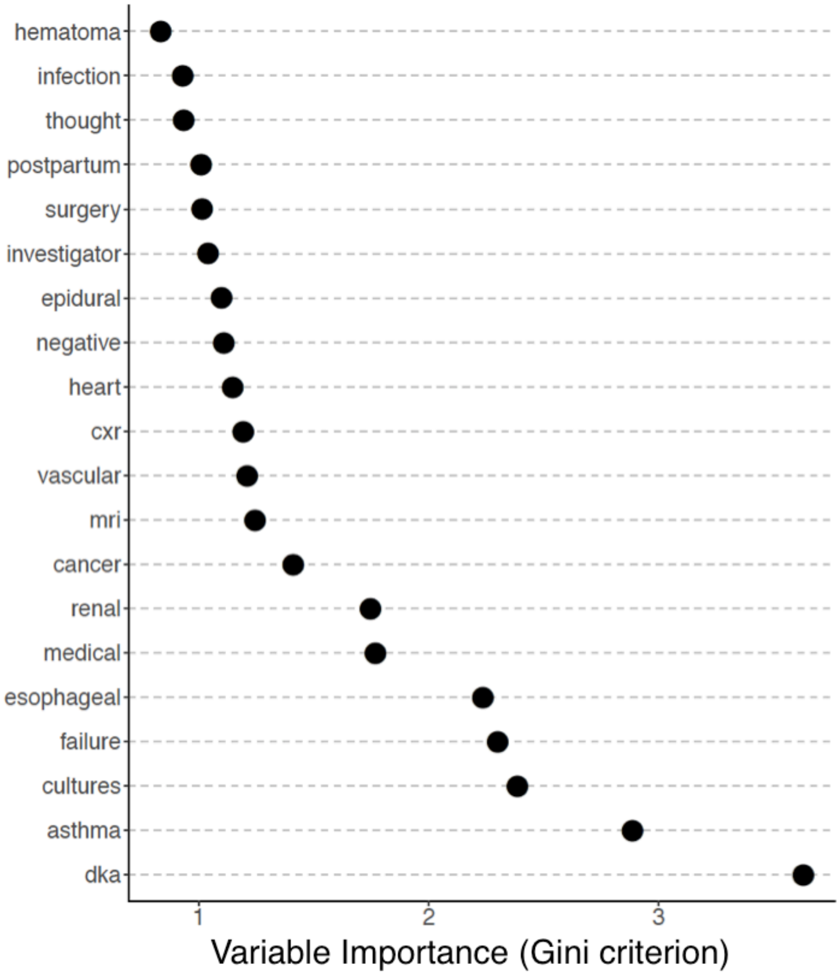

The next step in the linguistic analysis was to consider word-pairs in the same fashion. The Gini Index was used to rank the word-pairs in order of increasing importance (**Figure S7-4**), and the resultant ROC analysis gave AUC = 0.59 ± 0.08.

Figure S7-4: *Gini ranking of word-pairs in the 'physician comments' field of the case report form, for SIRS patients.* The ranking is based on contribution toward discriminating antibiotic-treated vs. untreated SIRS patients. Abbreviation: chf, congestive heart failure.

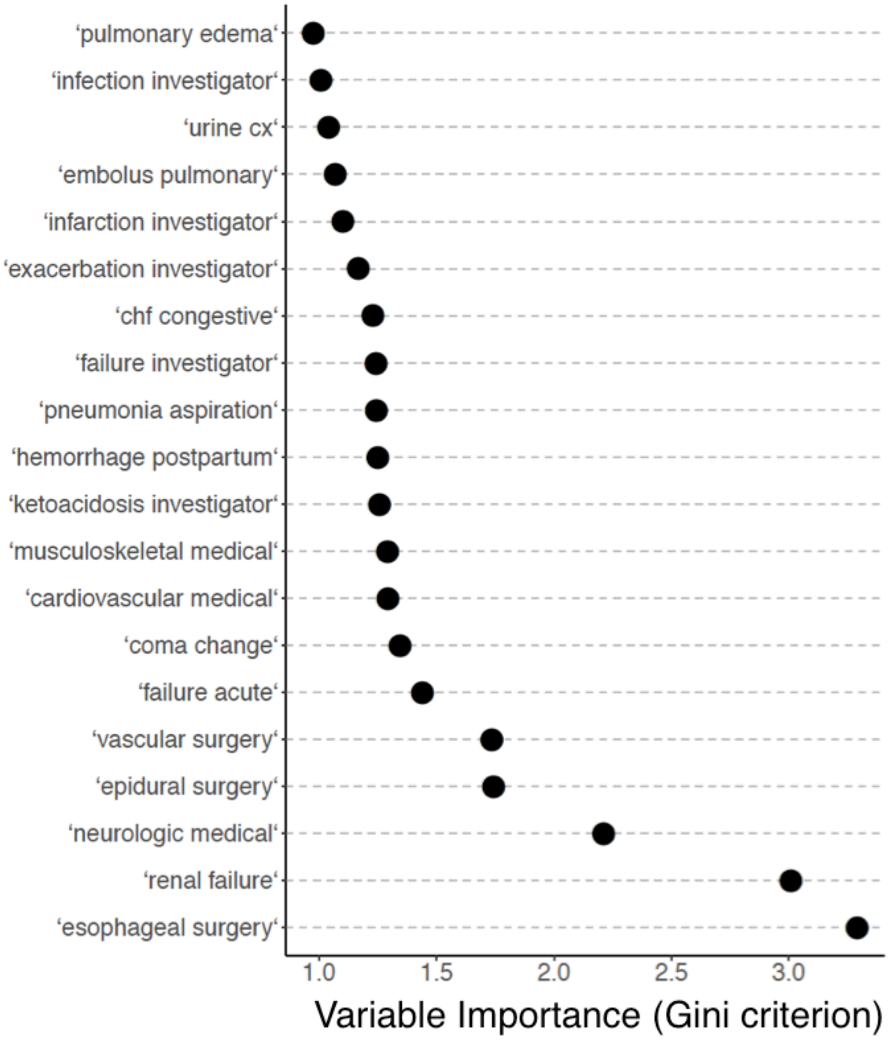

The final step in our machine learning exercise was to pool the most informative clinical and demographic parameters (Figure S7-2), words (Figure S7-3, Gini Criterion > 2.0), and word-pairs (Figure S7-4, Gini Criterion > 2.0), and then repeat the recursive variable elimination process with either logistic regression or Random Forests.

Results are shown in Figure S7-5. A marginal improvement was observed over the previous LR and RF models without the words or word pairs (compare Figure S7-2). However, under no conditions was the AUC much above a value of 0.7.

Figure S7-5: *Machine learning attempt to identify combinations of clinical variables, demographic variables, words and word-pairs that discriminate between antibiotic treatment and no treatment, within the SIRS group.* Recursive feature elimination was used, within a logistic regression (LR) or Random Forests (RF) model.

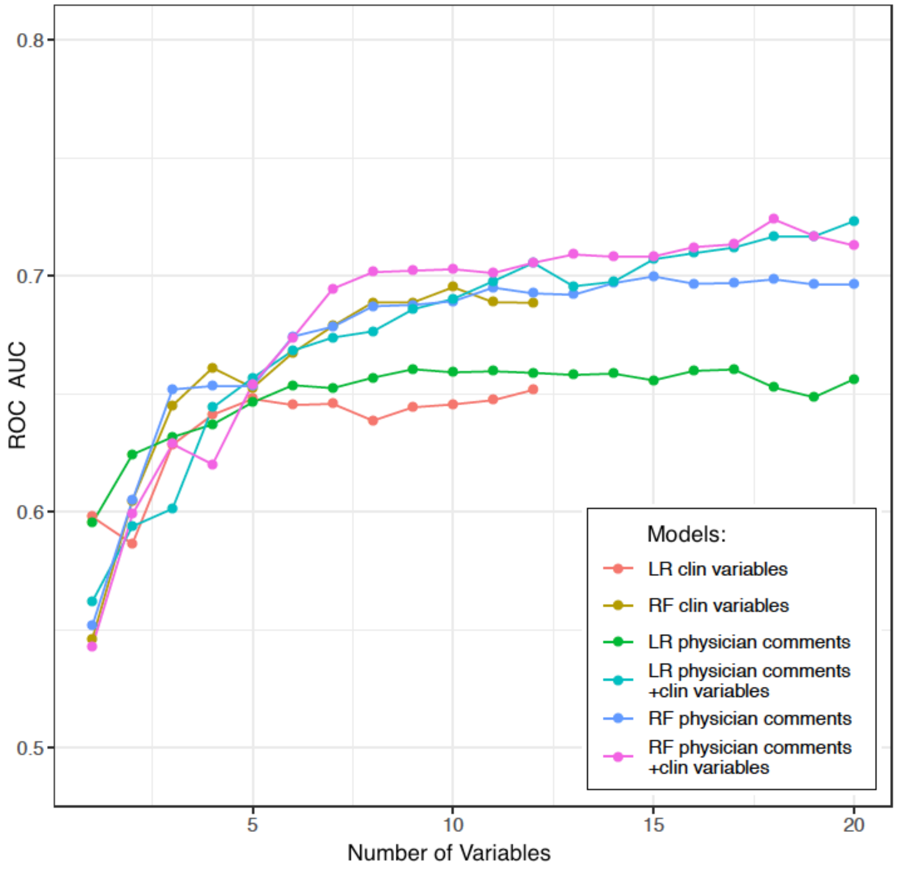

We conclude that the most informative combinations of these variables do convey some diagnostic information, contrary to the null hypothesis (H<u>o</u>) stated above. Nonetheless, we were not able to find any variable combinations that could with a high degree of accuracy separate the treated and untreated patients within the SIRS group.

References

Breiman L. (2001) Random Forests. J. Mach. Learn. 2001, 45: 5–32. doi: 10.1023/A:1010933404324.

Liaw, A. Documentation for R package randomForest (16 October 2012). Accessed at the following website on July 7, 2017: https://cran.r-project.org/web/packages/randomForest/randomForest.pdf.

Guyon I, Weston J, Barnhill S. Gene selection for cancer classification using support vector machines. Machine Learning 2002, 46: 389-422.

Kuhn, M. Documentation for the R package caret v6.0-81, algorithm rfe (Backwards Feature Selection). Date published: 2018-11-20 10:10:14 UTC. Accessed January 2, 2019 at the following website: https://www.rdocumentation.org/packages/caret/versions/6.0-81/topics/rfe.
